# INFLUENCE OF GLOBAL CLIMATE CHANGE ON CHEMICAL FATE AND BIOACCUMULATION: THE ROLE OF MULTIMEDIA MODELS

**DOI:** 10.1002/etc.2044

**Published:** 2012-12-18

**Authors:** Todd Gouin, James M Armitage, Ian T Cousins, Derek CG Muir, Carla A Ng, Liisa Reid, Shu Tao

**Affiliations:** † Unilever, Safety and Environmental Assurance CentreColworth Science Park, Sharnbrook, United Kingdom; ‡ Department of Occupational Medicine, Aarhus University Hospital, Aarhus C, Denmark, and Department of Physical and Environmental Sciences, University of Toronto–ScarboroughToronto, Ontario, Canada; § Department of Applied Environmental Science, Stockholm UniversityStockholm, Sweden; ‖ Aquatic Ecosystem Protection Research Division, Environment CanadaBurlington, Ontario, Canada; # Safety and Environmental Technology Group, Institute for Chemical and BioengineeringZurich, Switzerland; †† Canadian Environmental Modelling Centre, Trent UniversityPeterborough, Ontario, Canada; ‡‡ College of Urban and Environmental Sciences, Peking UniversityBeijing, People's Republic of China

**Keywords:** Climate change, Bioavailability, Persistent organic pollutant, Chemical space

## Abstract

Multimedia environmental fate models are valuable tools for investigating potential changes associated with global climate change, particularly because thermodynamic forcing on partitioning behavior as well as diffusive and nondiffusive exchange processes are implicitly considered. Similarly, food-web bioaccumulation models are capable of integrating the net effect of changes associated with factors such as temperature, growth rates, feeding preferences, and partitioning behavior on bioaccumulation potential. For the climate change scenarios considered in the present study, such tools indicate that alterations to exposure concentrations are typically within a factor of 2 of the baseline output. Based on an appreciation for the uncertainty in model parameters and baseline output, the authors recommend caution when interpreting or speculating on the relative importance of global climate change with respect to how changes caused by it will influence chemical fate and bioavailability. Environ. Toxicol. Chem. 2013;32:20–31. © 2012 SETAC

Changes to the abiotic and biotic components of the environment as a result of global climate change (GCC) may impact how we currently assess the environmental risks of chemicals 1–3. Environmental risk assessment requires an understanding of the relationship between exposure and effects, whereby exposure is typically estimated using an environmental fate model 4, 5. Alternatively, exposure can also be determined for specific locations based on measured concentrations.

A number of recent reviews have attempted to define how GCC will potentially influence chemical fate and bioaccumulation 1, 6–11. Several attempts have also been made to use computer models to project the impact of GCC on the fate and bioaccumulation of chemicals in future scenarios 12–19. In many instances, the reviews and modeling studies have focused on the Arctic and on either persistent organic pollutants (POPs) or mercury 6, 10, 11. Although we are aware that there is much work aimed at gathering empirical evidence for the impact of GCC on chemical fate and bioaccumulation, in the interests of brevity, we limit this contribution to reviewing the issue using modeling tools, with an emphasis on quantitatively defining the properties of chemicals most likely to be influenced by GCC, with respect to chemical fate and bioaccumulation.

We wish to initiate a tiered approach, whereby we begin our assessment at the global scale, with an emphasis on the factors influencing the fate of neutral organic chemicals. The approach we take in the present study largely utilizes available tools and draws on the expertise of the authors; therefore, it does not necessarily cover all possible scenarios or chemical classes. Consequently, this contribution should not be perceived as providing a definitive review of the influence of GCC on the chemical fate and bioaccumulation of all chemicals but as an illustrative example of how we might begin to address the issue.

Following a brief review of the state of knowledge regarding global climate model projections and scenarios, we derive new model calculations for determining how GCC may affect ambient environmental concentrations on a global scale and bioaccumulation. In these modeling approaches, we utilize chemical partitioning space plots to systematically determine which combination of chemical partitioning properties (air–water, octanol–water, and octanol–air partition coefficients [*K*_AW_, *K*_OW_, and *K*_OA_, respectively]) exhibit the greatest response to the GCC scenarios considered.

## CLIMATE MODEL PROJECTIONS AND SCENARIOS

**Fig. 1:**
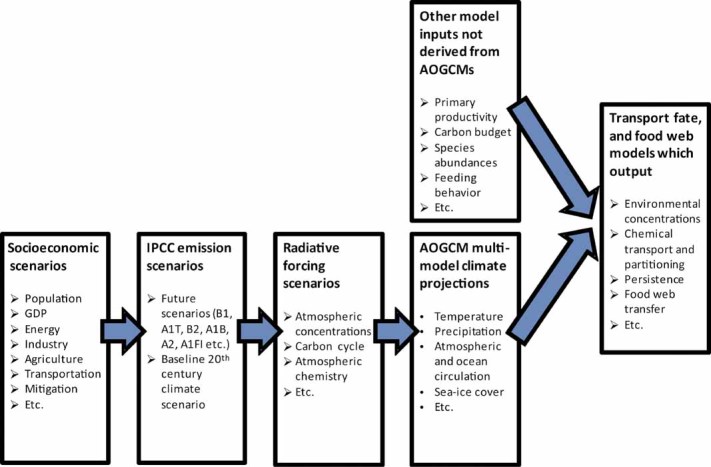
Sequential process involved in modeling global climate change impacts on fate and bioaccumulation. Considerable uncertainties are associated with each step in this process. Adapted from 25, 84. GDP = gross domestic product; IPCC = Intergovernmental Panel on Climate Change; AOGCMs = atmospheric-ocean general circulation models. [Color figure can be seen in the online version of this article, available at http://wileyonlinelibrary.com]

The GCC scenarios used in fate and transport models are typically developed from a linear sequential process that extends from the socioeconomic factors that influence emissions of greenhouse gases to atmospheric and climate processes to impacts on fate and bioaccumulation (Fig. 1). In each transition there are large uncertainties that propagate through to the climate model projections of the future. These uncertainties have been divided into the following three categories 20: (1) the internal variability of the climate system, that is, the natural fluctuations that arise in the absence of any altered radiative forcing of the planet; (2) model uncertainty in response to the same radiative forcing, that is, different atmospheric–ocean general circulation models simulate different changes in climate; and (3) uncertainty in future emissions of greenhouse gases. These uncertainties influence the uncertainty in projected impacts of GCC on the fate and bioaccumulation of chemicals. In addition, confidence in the changes projected by global models is known to decrease at smaller regional or local scales; consequently, other techniques, such as the use of regional climate models or downscaling methods, have been specifically developed 21. Uncertainties are also introduced if it is necessary to mathematically transform climate projection data from atmospheric–ocean general circulation models for use in fate and transport models to cater for differences in spatial and temporal resolution between different types of models.

In the Intergovernmental Panel on Climate Change (IPCC) Fourth Assessment report, the IPCC used greenhouse gas emissions and radiative forcing scenarios to drive multiple atmospheric–ocean general circulation models (>20 separate models) to make projections of future GCC (e.g., mean global temperature change, sea-level change, patterns of precipitation changes, changes in sea ice cover) and their potential impacts 22. For many climate scenarios, the report provides multiple-model projections for the six representative energy scenarios, described in the Supplemental Data. Projections can be quite variable between models and emission scenarios. We summarize some of the projections of GCC-related impacts and outline their associated uncertainties in Table 1.

**Table 1:** Global climate change (GCC) impact projections (at 2090–2099 relative to 1980–1999) from multiple model assessments undertaken by the Intergovernmental Panel on Climate Change (IPCC) and their associated uncertainties^a^

Selected parameters	GCC projections	Uncertainty judgment
Mean temperature change	+1.1 to +6.4°C	Consistent increase in temperature for all scenarios. The IPCC judges that hot extremes and heat waves will be more common.
Sea-level change	+0.18 to +0.59 m	The IPCC report states that these projections are highly uncertain because understanding of some important effects driving sea-level rise is too limited.
Precipitation change	−20 to +20%	Increases in the amount of precipitation are very likely in high latitudes, while decreases are likely in most subtropical land regions. Large disagreement between models for spatially specific projections in many regions. Increased incidence of storm events and flooding.
Ocean acidity	−0.14 to −0.35 pH units	Largest uncertainty in the future projection associated with the future projections of atmospheric CO_2_.
Sea-ice cover change	Sea ice is projected to shrink in both the Arctic and Antarctic under all emission scenarios. In some projections, Arctic late-summer sea ice disappears almost entirely by the end of the 21st century.	Consistent projection of decrease in Arctic and Antarctic sea-ice cover in all models, although exact amount varies largely between emission scenarios and models.
Ocean circulation	Increases and decreases in ocean currents and ocean circulation patterns. Zero change to more than 50% reduction in the Atlantic Ocean Meridional Overturning Circulation (MOC).	It is considered very likely that the MOC will slow down during the course of the 21st century. Models consistently predict this, although there is a large variation between models.
Wind fields and wind speed	Increases and decreases in mean wind speeds by 10–20% and changes in wind directions. Increased peak wind intensities and increased frequency of tropical storms.	Large uncertainties in predictions and high spatial variability.

a From Pachauri and Reisinger 22.

The outputs from the various models (Table 1) project that global mean temperatures will rise, ocean acidity will increase, and glaciers and other land ice will melt, resulting in a rise in sea levels. The magnitude of future changes for each of these parameters is, however, highly uncertain. Furthermore, although precipitation patterns will be altered, the direction of change varies with season and location, with larger uncertainties associated with projecting changes at the local or regional scale. It is therefore important to understand how uncertainty in these various environmental parameters might influence output obtained from environmental fate and transport models, as well as how changes due to GCC might influence the use and environmental release of chemicals.

### Estimating GCC impacts on chemical emissions

Chemical emissions are important input parameters to any multimedia fate model and strongly influence estimates regarding chemical fate. Unfortunately, information on emissions of chemicals used in commerce is limited due to a paucity of empirical information and lack of estimation methods, leading to large uncertainties associated with emissions 23. Here, we explore a number of illustrative scenarios regarding how estimated emissions of chemical contaminants may change as a result of changing rates of mobilization from materials and stockpiles, changing land-use patterns, migration of pests and infectious diseases, and stresses on forested systems due to decreased precipitation and increased temperatures that can trigger widespread forest fires.

### Impact of temperature on emissions

It is expected that GCC will lead to increased ambient temperatures, which could lead to increased emissions of chemicals through passive volatilization from materials and stockpiles 10, 15. In a recent study 15, the effect of temperature on the primary emission rate of polychlorinated biphenyl (PCB)-like compounds was estimated as a function of the internal energy of vaporization (dU_A_; see Supplemental Data) and shown to be more important than the effect of other GCC alterations on environmental fate, including the effect of temperature on revolatilization of PCBs from secondary sources (i.e., surface reservoirs). Alterations to passive volatilization from materials and stockpiles will be most relevant for chemicals with relatively low direct emissions during primary manufacturing, incorporation into materials (e.g., flame-retardant applications), and waste disposal (e.g., incineration). Consequently, the importance of GCC in this context will depend on the relative importance of different pathways throughout the chemical life cycle and thus does not lend itself well to simple generalizations.

### Changing land-use patterns

Emissions of current-use pesticides are likely to be altered under GCC as a result of changes in global agricultural practices. It is possible that GCC will affect yields and types of crops grown, thus influencing pesticide formulations used and application rates in specific regions 7. Changes in local weather patterns due to GCC can impact agriculture both positively and negatively. For instance, anticipated changes due to GCC, as summarized in Table 1, can affect the following: (1) the availability of potentially arable land 24, (2) the suitability of land for cultivation of specific crops, and (3) changes in crop yield. On a global scale, the change in potentially arable land is not expected to be large (−2 to +2% difference from the present, based on an ensemble of 13 global circulation models over two different energy scenarios 24); but the net effect, when factoring in population growth and residential and conservation land needs, can lead to greater projected losses (−2 to −9% on a global scale). In addition, regional-scale differences can be much greater. For example, the expansion of potentially arable land in northern zones such as the northern United States, northern China, and Russia can be as large as 17 to 56%, while the decrease at lower latitudes (e.g., Africa, Europe, South America) can be as large as 11 to 21% 24. Expansion of agriculture in northern areas can lead to substantial changes in crop type and seasonal practices, which could lead to potential shifts in both the type and timing of pesticide application 25.

### Vector control

Substantial uncertainty surrounds our current ability to project how the distribution of pests and infectious diseases will change as a result of GCC 26–37. For instance, indirect effects due to GCC that influence population growth, social and economic development, agricultural practices, and ecosystems, in combination with direct effects of GCC, such as increasing temperatures and changes to precipitation rates, may increase or shift the geographical distributions of pests and diseases. Projecting these changes, however, represents a major challenge 34, 38.

For example, a number of different types of insecticides are used as vector control for malaria, including DDT, pyrethoids, and malathion. These chemicals are used as part of various programs designed to combat the spread of malaria 39. Several studies, however, indicate developing resistance to pyrethroids and DDT by *Anopheles gambiae* and *Anopheles arabiensis*, the two most important malaria vectors across sub-Saharan Africa 40–43. Consequently, although the geographic ranges in which the dominant vectors for malaria may change as a consequence of GCC 37, ongoing activities aimed at monitoring insecticide resistance are essential to ensuring the implementation of an effective vector-control strategy.

While it is likely that distributions of pests and infectious diseases will be altered in the future, it is less clear what type of control strategies will be used to manage the change. These may include the use of nonchemical forms of control, such as integrated vector-control strategies aimed at reducing suitable habitats for the dominant vector species, or the use of novel chemical control, such as fungal biopesticides 44, 45.

### Energy use and forest fires

The demand for energy consumption is strongly influenced by changes in climatic conditions, with increases in demand correlated with extremes in temperatures 46. In addition to changes in temperature, factors influencing socioeconomic parameters can influence the demand and magnitude of energy consumption 47.

Global climate change, however, is also projected to increase the frequency of forest fires, which corresponds to elevated emissions of PAHs and other combustion by-products. The importance of these wildfire-mediated changes in emissions depends on the fraction of total emissions typically attributable to such events in a given year. In China, for example, estimated annual average emissions of PAHs due to forest and grassland fires are low (<10%) in comparison to emissions from intentional biofuel combustion 48, 49. Nevertheless, wildfire emissions can be influential over short periods (e.g., peak exposure levels), particularly at local to regional scales and in locations with low anthropogenic emissions. Better knowledge of wildfire emission factors and total emission inventories is required to assess the potential implications of GCC more comprehensively.

## APPLYING QUANTITATIVE TOOLS TO ESTIMATE GCC IMPACTS ON CHEMICAL FATE AND BIOACCUMULATION

### Environmental fate models

Currently, a variety of modeling tools can be used to investigate how GCC might influence the bioaccumulation of and exposure to chemical contaminants. Projections made using these models are proportional to emission rate, and hence sensitive to the assumed GCC-induced changes in emissions of chemicals reviewed in the last section, but will also be dependent on climate-induced changes to chemical transport, partitioning, and fate. Here, we summarize the current state of knowledge regarding how these tools have been utilized, to suggest potential impacts.

Fate and transport models are well-established tools for characterizing the behavior of contaminants in the environment. Fugacity-based multimedia fate models 50 are most commonly applied for neutral organic chemicals, but other models are available for ionizing chemicals 51–55 and some metals, including mercury 56–59. At the hemispheric to global scale, fate and transport models have been parameterized at a variety of spatial resolutions (e.g., broad latitudinal bands → 1 × 1° resolution) 60–64 and can be used for either steady-state or dynamic calculations.

As parameterization of such fate and transport models already encompasses factors such as temperature, precipitation, atmospheric circulation patterns, and degradation pathways/half-lives, modifying and applying these tools to investigate the potential influence of certain aspects of GCC is relatively straightforward. Indeed, several studies considering different climate or GCC scenarios ranging from the local to global in scale have already been published, albeit for a limited set of neutral organic chemicals (Table 2). The general approach is to define both a baseline and a future parameterization set and then generate and compare results for the two scenarios. Model output under the GCC scenarios considered to date is generally within a factor of 2 or less of the baseline results, with concentrations in air tending to be elevated under GCC but concentrations in other compartments tending to be lower in comparison 12, 14, 15. Results thus imply a relatively small influence of GCC on environmental concentrations.

The parameterization utilized, however, represents a gross simplification of the real environment. Model output from fate and transport models is expected to be best suited to characterizing long-term average patterns (spatially, temporally) and to be least suited to characterizing local-scale and/or rapid changes (e.g., extreme events). With respect to assessing the potential influence of GCC on contaminant fate, transport, and bioavailability, two key challenges illustrate the limitations of current fate models. First is the characterization of the Arctic and alterations that are projected or expected for this region. This is because changes in the Arctic due to GCC are projected to be significant, impacting a large spatial area and a variety of different environmental parameters. These changes include reduced sea-ice cover and altered sea-ice age structure (single vs multiyear), melting permafrost and glaciers, increased coastal erosion and carbon flows (i.e., large changes to biogeochemical cycling), and possible changes to vegetation cover 8. While sea-ice and snow-cover are included in some fate and transport models 65, 66, representations of other processes are not explicitly included, nor is parameterization of these processes likely to be a trivial exercise. The relative importance of these changes is thus difficult to ascertain and could vary depending on the emission phase of the chemical. For instance, it is anticipated that there may be a rerelease of legacy chemicals due to remobilization from reservoirs versus the release of currently used chemicals into the environment, whereby emissions may be influenced by long-range atmospheric and/or oceanic transport or due to regional use. Consequently, the uncertainties related to how to parameterize environmental fate and transport models to account for changes in emissions due to changes in human behavior, changes in biogeochemical cycling, changes in vegetation cover, melting permafrost, and so on, all represent considerable challenges that must be addressed to more comprehensively project the impact of GCC on the environmental fate and transport of chemicals.

A second key challenge is to incorporate changes to the organic carbon cycle, such as altered primary productivity or enhanced inputs of land-based organic carbon (e.g., melting permafrost), into aquatic environments. Such changes can be represented by altering the volume fraction of dissolved and particulate organic carbon in the water column and organic carbon contents of sediments or soils as proxies for such processes. Dachs et al. 67, for instance, applied modeling approaches to explore the role of eutrophication on air–water exchange, sinking fluxes, and biomass of phytoplankton, using PCBs in Lake Ontario as a case study. Interestingly, the authors concluded that the introduction of controls to reduce eutrophication could lead to increased concentrations of hydrophobic compounds in the pelagic food web (i.e., reduced phytoplankton biomass = reduced dilution effect = higher exposure), assuming constant atmospheric inputs to the aquatic ecosystem 67. These results are consistent with an earlier empirical study 68, which observed that variability in organochlorine pesticide concentrations in lake surface waters could be related to variability in planktonic biomass. In contrast, Carrie et al. 69 reported a positive relationship between increasing primary productivity, inferred from historical changes in lake sediment organic matter content, and concentrations of Hg and PCBs in burbot collected from the Mackenzie River. Additional research is thus recommended to more definitively elucidate the potential influence of changes in primary productivity due to GCC 70 on contaminant exposure in pelagic and benthic food webs. To date, however, there has been limited use of multimedia fate models to explore how changes in primary productivity related to GCC may influence the fate and bioavailability of chemicals. A key challenge relates to uncertainties in projecting how GCC will impact primary productivity, thus making it difficult to parameterize multimedia fate models accordingly 10. Additionally, the sorptive capacity of different organic carbon sources, as characterized by the organic carbon–water partition coefficient (*K*_OC_), may vary substantially. Nevertheless, models represent a powerful tool to better define the key processes that may influence exposure pathways in relation to changes in primary productivity.

### Bioaccumulation models

Tools available to investigate the effects of temperature on bioaccumulation largely derive from the combined application of bioaccumulation models that describe the uptake and distribution of chemicals in biotic systems with temperature-dependent chemical fate and bioenergetics models 71, 72. In addition, changes in trophic linkages can be considered, via changes in food-web structure for biota or, more empirically, by consideration of different diet compositions for human populations.

To date, only a limited number of modeling studies have addressed the interaction of climate and bioaccumulation, and most have focused primarily on one aspect (e.g., chemical distribution, species metabolic rates) and/or a single region (e.g., Arctic, Great Lakes). The interactions between climate and bioaccumulation potential were explored by Undeman et al. 73 through the use of linked chemical fate and food-web bioaccumulation models (CoZMoPOP2 and ACC-HUMAN) that described the exposure of humans with different diets in different hypothetical ecoclimatic zones to a hypothetical suite of chemicals spanning a range of physical–chemical properties (characterized by *K*_OA_ and *K*_OW_). In this study, the diet of human populations, which does vary with ecoclimatic region, rather than explicit links between climate parameters (e.g., temperature, precipitation) and chemical fate and transport, had the largest influence on bioaccumulation 73.

Using temperature-dependent uptake and loss rates in an aquatic bioaccumulation model, Borgå et al. 16 simulated the effect of climate change on bioaccumulation in an Arctic marine pelagic food web. Climate was modeled in two respects: (1) temperature, which was incorporated into the bioaccumulation model through the Q10 approach to changing respiration, consumption, and growth rates; and (2) primary productivity, which was assumed to increase, resulting in increasing concentrations of particulate organic carbon in the water column 16. For each chemical considered, a decrease in bioaccumulation was predicted 16. In cod, the species most strongly affected, changes ranged from being negligible to at most a 50% decrease 16. These decreases were controlled not by temperature-mediated uptake and loss rates within the organisms but primarily by reduced bioavailability resulting from partitioning of the chemical from the water column to the larger mass of particulate organic carbon (due to the assumed increase in primary productivity) 16.

Ng and Gray 17 focused their investigation of bioaccumulation and climate change on the impact of projected temperature increases in the Great Lakes on the metabolic rates of fish with different thermal sensitivities. Their study considered only PCB-77 and did not consider changes in the productivity or organic carbon content of the water column. However, bioenergetics models for three fish species were parameterized with species-specific thermal relationships for consumption, respiration, and growth. Consumption and growth rates, which are positively correlated in bioenergetics models but negatively correlated in bioaccumulation models, were predicted to have a balancing effect on the influence of temperature on chemical concentrations in biota. Changes in PCB-77 concentrations in fish due to projected temperature shifts in Lake Erie and Lake Superior were relatively small, particularly when compared with potential changes in food availability and food type 17. Metabolic effects (temperature effects on the rate of chemical uptake and loss) were confounded by trophic interactions among species with different thermal sensitivities, indicating possible effects of invasions of warm-water species into warming higher-latitude territories 17.

Although these three modeling exercises all focused on different aspects of the effect of climate on bioaccumulation, a clear and consistent finding emerged from each of them—the major impact of climate on chemical exposure for both humans and biota is likely to be from indirect effects, such as food-web changes (e.g., primary productivity, species invasions, changes in human diets), as opposed to the direct bioenergetic impacts of temperature on uptake through the consumption rate or on loss processes such as growth dilution. Unfortunately, indirect effects are the least defined from both a modeling and an empirical data standpoint. Thus, a better understanding of the effects of climate change on species distributions and trophic interactions is needed for improving our understanding of the most likely and most important impacts of GCC on bioaccumulation 74.

## CHEMICAL PARTITIONING SPACE PLOTS

As described in the present study and elsewhere 3, 8, there are many processes and interactions related to GCC with the potential to influence the fate and transport of chemicals in the environment at different spatial and temporal scales. The behavior of chemicals in the environment, however, is strongly influenced by their physical–chemical properties and, consequently, so is the sensitivity to various perturbations associated with GCC.

In an effort to quantify the physical–chemical properties most sensitive to changes due to GCC, we utilized a chemical space approach, whereby a large number of model simulations, based on a set of hypothetical persistent chemicals covering a wide range of possible combinations of partitioning behavior (see Supplemental Data), are performed. Model output is then summarized in the form of chemical space plots 75. Such an assessment provides information on the directionality and magnitude of the influence of these alterations, which can then serve as the background context against which other potential changes related to GCC can be compared. A key consideration of the model parameterization relates to the temperature dependence of degradation reactions in the environment as well as the physical–chemical properties of the chemicals, especially when comparing model output between different climates. The activation energies for degradation in different environmental media assumed for all hypothetical chemicals and the approach adopted for their partitioning properties are summarized in the Supplemental Data. Briefly, the internal energy of the phase change for octanol–water partitioning (dU_OW_) is defined as −20 kJ/mol, which is consistent with values reported for a number of known POPs 76. The enthalpies of phase change for octanol–air partitioning (dU_OA_) are described in Supplemental Data and that for air–water partitioning (dU_AW_) has been defined in a manner to ensure internally consistent property data (i.e., dU_OW_ – dU_OA_ 76).

### Global-scale fate and transport simulations

The main purpose of the global-scale fate and transport simulations conducted here is to identify broad patterns in the response of different environmental compartments (e.g., air, water, soil) to changes in thermodynamic forcing (e.g., temperature dependence of environmental partitioning and degradation reactions) and precipitation scavenging (an important factor in establishing long-range transport potential for some compounds). Based on how the model has been parameterized, these simulations are most relevant to neutral organic compounds, but the approach described here could also be adopted to include ionogenic organic chemicals (e.g., 51–55).

A novel version of the multimedia environmental fate and transport BETR-World model 77 was used for these simulations (Supplemental Data). A fugacity-based fate and transport model, BETR-World represents the globe as a series of interconnected regions, each subdivided into bulk compartments representing the different environmental media present (e.g., atmosphere, freshwater, sediments, soil, vegetation, surface ocean water). The model has been revised to match the geographical units for which regional climate projections (temperature and precipitation change) were generated by the IPCC 22 and is therefore referred to here as BETR-IPCC. Additional details regarding model parameterization and application are provided in the Supplemental Data.

**Fig. 2:**
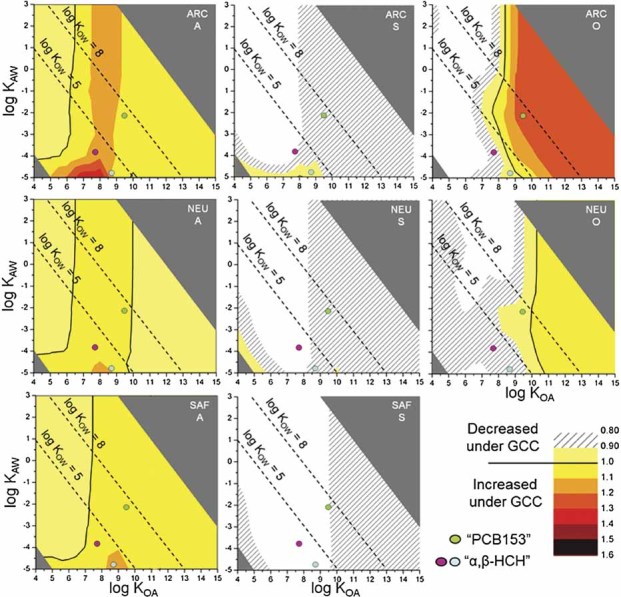
Comparison of steady-state surface air (A), soil (S), freely dissolved freshwater, sediment pore-water, and freely dissolved surface ocean concentrations in the Arctic (ARC), northern Europe (NEU), and South Africa (SAF) model regions of BETR- Intergovernmental Panel on Climate Change under the global climate change (GCC) scenario compared to the baseline scenario (presented as the ratio of GCC model output to baseline model output). Partitioning properties for polychlorinated biphenyl (PCB)-153 and α- and β-hexachlorocyclohexanes (25°C) are also shown.

Briefly, projections for temperature and precipitation changes for the A1B scenario for the period 2080 to 2099 were summarized for 30 regions in the IPCC report 22 on a seasonal and annual basis and then compared to the baseline period, 1980–1999. The regional identifiers are presented in Supplemental Data and the projections for each region for the A1B scenario based on 21 models are presented in Supplemental Data (annual basis). Figure 2 summarizes the chemical space plots produced from the BETR-IPCC model, comparing output for three different geographical regions. Concentration ratios (GCC to baseline) are presented for the lower atmosphere and soil solids, as are freely dissolved concentrations for surface ocean water. Freely dissolved concentrations in the aqueous environments were selected because they are the most relevant for exposure in pelagic food webs. As a reference point, the majority of known POPs occupy the region defined approximately by log *K*_AW_ = −1 to −5 and log *K*_OA_ = 7 to 13.

All model outputs under the GCC scenario are well within a factor of 2 of the output from the baseline scenario across the entire chemical space considered here (and typically ± 20%) (Fig. 2). One broad response across the chemical space is that concentrations are projected to increase in the surface air compartment and projected to be reduced in the bulk soil compartment. Concentrations of dissolved compounds are projected to exhibit both negative and positive responses to the GCC scenario, with more positive responses exhibited by the more hydrophobic chemicals (lower right side of panels shown in Fig. 2). The Arctic environment is projected to be somewhat more sensitive to changes introduced in the GCC scenario, as can be seen in the results for the lower atmosphere and concentrations dissolved in marine waters. Given that the model simulation assumes that emissions to this region are very low, responses to the GCC scenario are thus sensitive to alterations in long-range transport to the region in addition to changes in processing/redistribution within the region.

Interestingly, the area of the chemical space showing an elevated positive response to the GCC scenario for surface air concentrations (i.e., log *K*_AW_ = −4 to −5, log *K*_OA_ = 6 to 9, Arctic [ARC] region) is similar to the partitioning properties of the hexachlorocyclohexanes (HCHs), which were among the group of chemicals recently suggested to be exhibiting climate change–induced alterations in environmental fate in the Arctic (enhanced revolatilization from surface reservoirs, attenuation of decline in atmospheric levels over recent decades) 18. Other POPs suggested to be affected 18 (e.g., chlordane, DDT) have partitioning properties corresponding to elevated surface air concentrations in the Arctic as well. It is important to note, however, that the pattern of responses in Arctic surface air in this region of the chemical space is not identical to other model output, demonstrating that behavior in one environmental compartment may not be representative of changes elsewhere.

As an additional point of reference, we also generated output for a chemical with the partitioning properties shown for PCB-153 in Figure 2 and with the degradation half-lives and temperature dependencies specific to this chemical. In general, the results are consistent with the main conclusions reported by Lamon et al. 15, using a similar modeling approach, namely, that the concentrations of PCB-153 in the lower atmosphere are elevated globally under warmer conditions but that overall persistence in the environment (*P*_OV_) is reduced (Table 2). Assuming no change to primary emission rates under the GCC scenario (e.g., due to temperature dependence of passive volatilization from stockpiles), the BETR-IPCC model projects that while concentrations in the atmosphere may be elevated (≍5–30%), the total global mass inventory is reduced (≍20–25%). In other words, as in Figure 2, changes in the lower atmosphere due to GCC do not reflect patterns and trends in other environmental media. This output also suggests that the magnitude of change in temperature (favoring the gas phase) is more influential than increased precipitation rates (favoring air-to-surface deposition) in determining the overall environmental fate of such compounds.

The key message of the output illustrated in Figure 2 implies that the long-term forcing on contaminant fate, transport, and bioavailability related to temperature and precipitation changes expected under GCC appears to be limited in magnitude and that this conclusion is valid across a wide range of physical–chemical properties. Other factors, such as changes in emission rate over time and proximity of sources to receptor sites of interest are likely to be far more influential on the evolution of contaminant burdens in the global context. Finally, surface air concentrations for neutral organics may not be a suitable proxy for characterizing the potential implications of GCC on ecological and human exposure.

### Bioaccumulation modeling

**Fig. 3:**
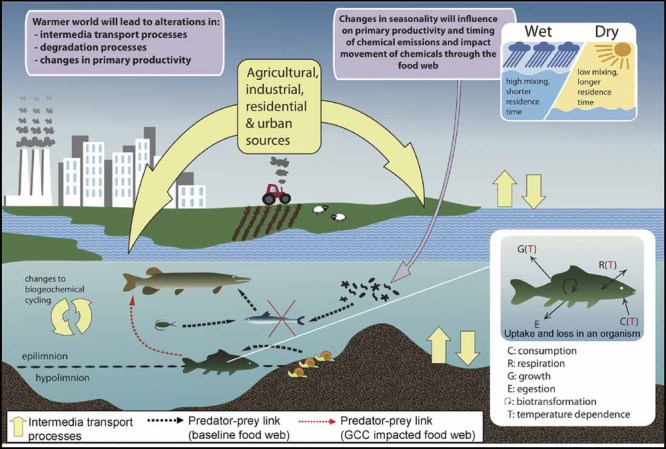
Influence of global climate change (GCC) on bioaccumulation at different scales.

Global climate change affects the bioaccumulation of contaminants through three primary mechanisms: environmental exposure (driven by concentrations encountered in surrounding media, Fig. 3), dietary exposure (driven by concentrations in food and the predator–prey connections that constitute the food web), and uptake and loss rates in organisms (driven by bioenergetic processes that control consumption, respiration, and elimination rates; Fig. 3 inset).

As discussed earlier, direct impacts of GCC may increase emissions of volatile chemicals and increase temperature-dependent degradation rates. However, indirect effects (such as regulation) may lead to decreases in primary emissions, and changing environmental conditions, such as to oxic/anoxic zones in aquatic systems, may lead to either net increases or decreases in degradation. Potential changes in food-web structure, which affect bioaccumulation via exposures encountered in the diet, can at times be difficult to predict, resulting in challenges with respect to integrating biological information across many organisms at different trophic levels 78. The within-organism impacts of GCC due to changes in bioenergetics rates have been previously explored by Ng and Gray 17 for several species and a single chemical. Their methods are expanded here (see Supplemental Data for details) to explore the potential impacts of GCC on the bioaccumulation in an organism of a wide range of chemicals.

**Fig. 4:**
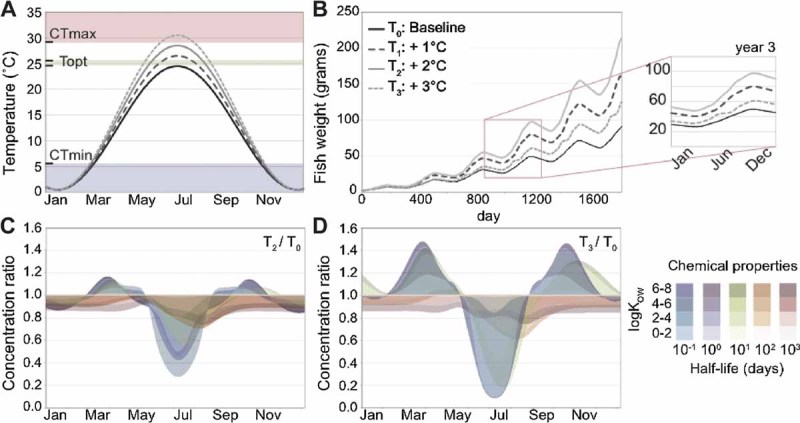
Impacts of global climate change on bioaccumulation within an organism. Species thermal range interacts with temperature scenarios (**A**) and with bioenergetics to affect growth (**B**), leading to highly seasonal bioaccumulation patterns that can be above or below baseline values (**C**, **D**).

The impact of GCC on bioaccumulation through bioenergetics depends largely on how annual temperatures relate to an organism's temperature preference and critical thermal limits. These can be broadly classified into three regions, illustrated in Figure 4A for the round goby (*Apollonia melanostomus*) 17: (1) the lower critical thermal limit (CT_min_), (2) the optimal temperature(s) for growth (T_opt_), (3) and the upper critical thermal limit (CT_max_). In terms of maximizing consumption and growth, the longer time spent near T_opt_, the better. Crossing either CT_min_ or CT_max_, on the other hand, causes a steep decline in these rates. Here, differences in round goby growth are illustrated based on a typical Lake Erie annual temperature cycle compared to a 1, 2, or 3°C increase in the annual average temperature (Fig. 4A; see Supplemental Data for details). Different annual temperature profiles can result in substantial differences in growth over the lifetime of an organism (Fig. 4B), driven largely by the acceleration and deceleration of growth at winter lows and summer highs (Fig. 4B, year three growth comparison). Thus, increasing annual average temperatures only increases growth when temperatures remain below CT_max_ (compared to the Lake Erie baseline scenario, an increase of 1 to 2°C results in substantial increased growth); but above a certain threshold, the benefits decline (at a 3°C increase, growth drops closer to the baseline case). Of course, the shape of these curves will vary according to the thermal limits and sensitivities of an organism, and impacts from GCC will depend in large part on whether organisms are at the colder (below T_opt_) or warmer (close to CT_max_) limits of their thermal range. Thus, the determining factor for the influence of GCC on an organism will not be the absolute change in temperature alone but rather how the change in temperature interacts with the species' thermal niche, that is, how close to its critical upper or lower thermal limits it is in the environment undergoing climate change.

We investigated the impacts of these temperature changes on bioaccumulation potential by considering the ratio of concentrations in the baseline and warmer scenarios, predicted in a round goby over the course of one year (year three in a 5-year simulation). We focus on the 2 and 3°C increases, which represent the fastest growth and highest temperature scenarios, respectively (Fig. 4C and D). Concentration ratios >1 indicate more bioaccumulation relative to the baseline case, whereas ratios <1 indicates less bioaccumulation. We do this for a set of hypothetical chemicals with biotransformation half-lives ranging from 0.1 to 1,000 d and log *K*_OW_ values ranging from 0 to 8.

We observe that seasonal effects are pronounced for both scenarios, with concentration ratios both above and below 1 in a single year. Increases in bioaccumulation relative to the baseline case occur during the spring and autumn, while decreases occur during the summer. This is an intuitive observation: when temperatures approach T_opt_, consumption rates increase. This causes an increase in uptake of the chemical with food. On the other hand, when summer temperatures exceed CT_max_, consumption rates, and thus uptake, fall.

The magnitude of the deviation from the baseline case depends most strongly on the biotransformation half-life and then on *K*_OW_. The strongest impact on bioaccumulation potential is on chemicals with biotransformation half-lives between 0.1 and 1 d (Fig. 4C and D) and log *K*_OW_ >6. Spring and autumn increases in bioaccumulation of the T3 scenario can be as high as 50% in concentrations relative to the baseline for these chemicals. More substantially, the summer decreases for this scenario can be dramatic, as large as a factor of 10. Reaching or exceeding CT_max_ can thus strongly influence chemical uptake and loss but only for those chemicals that undergo rapid biotransformation and thus are not bioaccumulative.

Interestingly, for what we might consider the classical POP-like chemicals (with log *K*_OW_ between 6 and 8 and half-lives >100 d), changes are much less pronounced seasonally, with maximum increases of only 10% and decreases of 20 to 40% (Fig. 4C and D). Thus, for chemicals that are considered to be problematic from a bioaccumulation perspective (i.e., persistent chemicals with low biotransformation rates) the direct influence of GCC through temperature-dependent uptake and loss rates is likely to be minimal. It would be of interest, however, to expand this assessment to chemicals of emerging concern.

It should be noted that projected changes in these simulations consider constant concentrations in the environment. We must also consider the overall landscape of uncertainty concerning chemical fate under GCC. First, there is the magnitude of uncertainty in projected temperature increases (Table 1) relative to the sensitivity of bioaccumulation to temperature changes (e.g., Fig. 4C vs D). Second, other GCC impacts will influence contaminant transport and thus the levels encountered by organisms in the environment, including changes in primary emissions, precipitation, and runoff. Resulting increases or decreases in environmental concentrations could be confounded by the seasonal variability in uptake and loss rates illustrated here (Figs. 4C and 5D), particularly for less persistent chemicals. Third, it is likely that species coexisting in a food web will have different thermal ranges and sensitivities, particularly as they adapt to climate warming. Thus, modeling the changes in bioaccumulation due to GCC in a way that can integrate physicochemical, ecological, and physiological impacts will be particularly challenging.

## THE ROLE OF UNCERTAINTY

To date, there has been little attention focused on the role of how uncertainty in model inputs can affect the ability to project GCC-induced impacts on fate and bioaccumulation. In the previous sections, we have illustrated that large uncertainties exist in GCC projections (e.g., annual mean temperature and precipitation; Table 1) and their secondary impacts on ecosystems (e.g., changes in primary productivity and feeding relationships in food webs). Although the projected variability in climate parameters is considered high and may have potentially devastating impacts on ecosystems, it remains unclear if it is possible to accurately predict GCC-induced impacts on chemical fate and bioaccumulation, which are clearly distinguishable from the noise generated by uncertainties in chemical inputs and in short-term climate variations (e.g., weakening of the North Atlantic Oscillation, which occurred in the late 1990s 13).

The relative importance of the sensitivity from the two main types of inputs in fate and bioaccumulation models—namely, landscape property inputs (environmental inputs including climate inputs) and chemical property inputs (physical–chemical properties, degradation rates, and emission rates)—has been determined previously in numerous modeling studies 79–82. In one of the earliest studies, Hertwich et al. 79 performed a sensitivity analysis on the influence of physical–chemical and landscape properties and exposure parameters on the variance in predicted exposure dose. They concluded that landscape properties contribute to 10% or less of the total variance when estimating potential doses of chemicals to humans 79. This has also been found in other studies for a range of additional model outcomes. For example, Fenner et al. 81 determined that persistence and long-range transport potential were mainly determined by chemical properties, and Hollander et al. 82 showed that the predicted multimedia concentrations for a set of 137 chemicals are mainly controlled by their substance-specific partitioning properties and degradation rates.

Climate properties typically used by modelers when projecting the impact of GCC on fate and exposure are a subset of the landscape properties, namely, mean temperature and mean precipitation (Fig. 1). Based on the literature review summarized in Table 2 and the results shown above in the chemical space plots (Figs. 2 and 4), it is suggested that the GCC scenarios considered can potentially alter predicted concentrations in exposure media at most by up to a factor of 2 for neutral organics. However, uncertainties resulting from uncertain physical–chemical and degradation inputs are at least a factor of 10 for any given exposure assessment and could potentially be much higher for chemicals with properties that fall outside the applicability domain of multimedia fate models.

**Table 2:** Summary of recent multimedia fate and bioaccumulation model output incorporating long-term GCC scenarios^a^

Study	Scale scope	Chemical compound	Changes	Results
McKone et al. 12	Regional (W. USA), SS fate model	HCB	T↑ (mean 2.5°C) Δ precipitation Δ wind speed (as fΔT)	Mean cancer risk ↓ (22%)
Macleod et al. 13	Global, SS fate model	PCB-28 PCB-153	NAO index used to scale changes in T and wind speed	C_AIR_↑ by max 2-fold with high NAO index under current extent of variability
Valle et al. 14	Local (Venice Lagoon), D fate model	PCB-118 PCB-180 TCDF HCDF	Emissions ↓ (10-fold, 50 years) T ↑(1, 3°C) Precipitation ↓ (5, 10%) Degradation ↑ (10, 30%)	C_AIR_ ↑∼10% C_SED_ ↓ 20–45% C_WAT_ ↓ 2–10% C_SPM_ ↓ 20–50% vs control at the end of 50-year simulation (i.e., ↑dissipation)
Lamon et al. 15	Global, SS fate model	PCB-28 PCB-153	A2 vs 20CE T↑ (∼1–8°C) Emissions ↑(fΔt) Δ Precipitation Δ Wind speed Δ Ocean currents	(1) C_AIR_ in Arctic ↑ by ∼2.0- to 2.5-fold; ↑emissions is the main factor (2) *P*_OV_ ↓
Ma and Cao 19	Closed two-compartment air-surface system, D fate model, perturbation approach	α-HCH γ-HCH HCB PCB-28 PCB-153	T↑ 0.05–0.1 K year^−1^ (air–soil system) Precipitation ± 20% (HCHs only, air–soil system)	4–50% increase in air concentration compared to mean ± 4 and ± 53% change from mean air concentration for α- and γ-HCH, respectively
Borgå et al. 16	Regional Arctic, SS bioaccumulation model	γ-HCH PCB-52 PCB-153	T ↑(2, 4°C) *f*_LIPID_ ↓ *f*ΔT ↓ (10, 20%) POC/DOC ↑ (50, 100%)	C_FISH_ ↓ vs control γ-HCH: *F*_MAX_ = 0.78–0.93 PCB-52: *F*_MAX_ = 0.44–0.62 PCB-153: *F*_MAX_ = 0.33–0.44
Ng and Grey 17	Regional (Great Lakes), D coupled bioenergetics/ bioaccumulation model	PCB-77	T ↑ (5–6°C) based on 100-year projections for Lake Superior surface water temperatures	C_FISH_ ↑ vs control species-specific and confounded by predator–prey dynamics *F*_MAX_ = approx 3

a Expanded from Armitage et al. 6.GCC = global climate change; HCH = hexachlorocyclohexane; fΔT = the parameter varied as a function of temperature; PCB = polychlorinated biphenyl; NAO = North Atlantic Oscillation; D = dynamic (non-steady state) simulation; A2 vs 20CE = use of climatic conditions modeled for the year 2100 under the A2 scenario compared to current conditions (see Borgå et al. 16 for details); *P*_OV_ = overall persistence in the environment; *F*_MAX_ = maximum factor of change (i.e., ratio) between model output under the GCC scenario compared to the default assumptions (e.g., *F*_MAX_ = 0.5 means GCC scenario output is twofold lower); HCB = hexachlorobenzene; POC = particulate organic carbon; DOC = dissolved organic carbon; TCDF = tetrachlorodibenzofuran; HCDF = hexachlorodibenzofuran.

McKone et al. 12 first considered the issue of how uncertainty about GCC could impact the precision of predictions of secondary outcomes such as the health impacts of pollution. Using a model that linked GCC with predictions of exposure to chemicals and human health risk in the western region of the United States, they defined parameter variability and uncertainties and characterized the resulting outcome variance 12. As a case study, McKone et al. 12 considered the public-health consequences from releases of hexachlorobenzene (HCB). Their assessment revealed that temperature increases of up to 5°C had little impact on either the magnitude or precision of the public-health consequences estimated for hexachlorobenzene (HCB). McKone et al. 12 observed that uncertainty in chemical inputs as well as existing seasonal climate variations had more impact on predicting human health outcomes than long-term changes in climate. In other words, the inherent uncertainty in baseline model output is a more important consideration in hazard/risk-assessment and risk-management decisions for a typical model application than the potential implications of GCC.

## CONCLUSIONS AND RECOMMENDATIONS

The following conclusions can be drawn from the literature review and model simulations conducted here. First, the effect of GCC on chemical emissions, which will have direct consequences for predicted exposure, is an important consideration, but the paucity of and uncertainty in current chemical emission inventories limit the ability to adequately quantify the impacts. Second, indirect effects, such as changes in species distribution and trophic interactions, changes to the organic carbon cycle, and characterization of regions strongly influenced by GCC, such as the Arctic, are difficult to parameterize but may have a more dramatic impact on chemical fate and behavior than will the influence of GCC on the physical–chemical properties of chemical compounds. Third, assuming unchanging and similar emissions for all chemicals, the multimedia modeling undertaken here demonstrates that the effects of the GCC scenarios considered on long-term chemical transport, partitioning, and fate are relatively small (i.e., within a factor of 2 of baseline output) for persistent neutral organics. The direct GCC-induced effects on bioaccumulation potential in an aquatic food chain vary substantially depending on partitioning properties and biotransformation rate constants. For neutral organic chemicals typically considered problematic with respect to bioaccumulation potential (e.g., POPs), responses are relatively small (i.e., within a factor of 2 of the baseline output). Fourth, the uncertainty in baseline model output related to chemical inputs (e.g., emissions, physical–chemical properties, degradation rates) for a given hazard/risk assessment is likely to be substantially larger than alterations in model output associated with the GCC scenarios. Lastly, expanding the applicability of the chemical space to quantify the potential influence of GCC on the chemical fate and bioavailability of ionizable organics and elemental contaminants represents an additional area of research needed in future exercises.

After consideration of the many sources of uncertainty, we conclude that caution is needed when evaluating the role of GCC, based on output obtained from multimedia fate and bioaccumulation models, as the cause–effect linkage of observed phenomena, such as changes in temporal trends 83. It would thus be unwise to rely solely on output from environmental fate and bioaccumulation models as indicators for defining the influence of GCC on exposure. Consequently, it is fundamental that strong time series for chemicals in biotic and abiotic media be developed and that research be directed toward detecting climate-related effects. We thus recommend a complementary approach that utilizes information obtained from both multimedia models and monitoring data.

## SUPPLEMENTAL DATA

**Tables S1-S10.**

**Figs. S1-S9** (5.9 MB DOC).
