# Supplementary material for: INFLUENCE OF GLOBAL CLIMATE CHANGE ON CHEMICAL FATE AND BIOACCUMULATION: THE ROLE OF MULTIMEDIA MODELS
Source: Environ Toxicol Chem. 2012 Dec 18;32(1):20–31. doi: 10.1002/etc.2044 (PMC3601418; doi:10.1002/etc.2044)
Supplement: Supplementary file 1 [file etc0032-0020-SD1.doc]

**Influence of global climate change on chemical fate and bioaccumulation: The role of multimedia models**

Todd Gouin†*, James M. Armitage‡, Ian T. Cousins§, Derek C.G. Muir||, Carla A. Ng#, Liisa Reid††, Shu Tao‡‡

† Unilever, Safety & Environmental Assurance Centre, ColworthSciencePark, Sharnbrook, MK441LQ, United Kingdom

‡ Department of Occupational Medicine, Aarhus University Hospital, Noerrebrogade 44, Aarhus C, Denmark 8000; Department of Physical and Environmental Sciences, University of Toronto – Scarborough, 1265 Military Trail, Toronto, Ontario, Canada M1C1A4

§ StockholmUniversity, Department of Applied Environmental Science (ITM), Frescativägen 8, Stockholm, SE-10691, Sweden

|| Environment Canada, Aquatic Ecosystem Protection Research Division, 867 Lakeshore Road, Burlington, ON L3M4E7, Canada

# Safety and Environmental Technology Group, Institute for Chemical and Bioengineering, ETH Zurich, Wolgang-Pauli-Strasse 10, 8093 Zurich, Switzerland

†† Canadian Environmental Modelling Centre, TrentUniversity, Peterborough, Ontario, CanadaK9J 7B8

‡‡ College of Urban and Environmental Sciences, Peking University, Beijing 100871 China.

Corresponding author:

Todd Gouin

Safety & Environmental Assurance Centre

ColworthSciencePark

Sharnbrook

Bedford

MK44 1LQ

UK

Tel. +44 (0)1234 264770

Fax +44 (0)1234 264744

todd.gouin@unilever.com

**Supplementary Information**

***Section S1: Defining GHG emission scenarios*:**

Future GHG emission scenarios have been derived by the IPCC and described in the IPCC Special Report on Emissions Scenarios [1]. These are subject to re-evaluations as understanding of the driving forces of emissions improves [2]. New approaches for constructing scenarios are under development [3]. Future GHG emissions are the product of very complex dynamic systems, determined by driving forces such as demographic development, socio-economic development, and technological change. Scenarios are alternative images of how the future might unfold and are used to analyse how driving forces may influence future emissions. Briefly, four different narrative storylines were developed to describe the relationships between emission driving forces and their evolution and add context for the scenario quantification [1]. Each storyline represents different demographic, social, economic, technological, and environmental developments. All the scenarios based on the same storyline constitute a scenario “family”; termed A1, A2, B1 and B2. The A1 storyline describes a world of very rapid economic growth, a global population that peaks in mid-century and rapid introduction of new and more efficient technologies. A1 is divided into three alternative directions of technological development: fossil intensive energy resources (A1FI), non-fossil energy resources (A1T) and balanced energy resources (A1B). B1 has the same global population as A1, but with more rapid changes in economic structures toward a service and information economy. B2 describes a world with intermediate population and economic growth, emphasising local solutions to economic, social, and environmental sustainability. A2 describes a world with high population growth, slow economic development and slow technological change. In total there are 40 distinct emission scenarios within these four families and all are considered equally valid with no assigned probabilities of occurrence. The IPCC AR4 [2] recommends that a range of GHG emission scenarios with a variety of assumptions regarding driving forces be used in any analysis of GCC impacts. As many as 6 marker scenarios are recommended [2], namely B1, A1T, B2, A1B, A2 and A1FI. It is not uncommon, however, for scientists modelling GCC impacts to consider fewer emission scenarios than recommended by IPCC (e.g. two opposite extreme emission scenarios; a high and a low GHG emission scenario are commonly used). Although considering several GHG emission scenarios would provide representative analysis, there can be practical problems for fate and bioaccumulation modellers involved in running vast numbers of model simulations.

**Table S1.** GCC impact projections (at 2090-2099 relative to 1980-1999) from multiple model assessments undertaken by IPCC and their associated uncertainties. (IPCC, 2007).

| **Selected parameters** | | **GCC projections** | | **Uncertainty judgement** |
| --- | --- | --- | --- | --- |
| Mean temperature change | | +1.1 to +6.4°C | | Consistent increase in temperature for all scenarios, IPCC judges that hot extremes and heat waves will be more common |
| Sea level change | | +0.18 to +0.59 m | | IPCC report states that these projections are highly uncertain because understanding of some important effects driving sea level rise is too limited |
| Precipitation change | | -20 to +20 % | | Increases in the amount of precipitation are very likely in high-latitudes, while decreases are likely in most  subtropical land regions. Large disagreement between models for spatially-specific projections in many regions. Increased incidence of storm events and flooding. |
| Ocean acidity | | -0.14 to -0.35 pH units | | Largest uncertainty in the future projection associated with the future projections of atmospheric CO_2_. |
| Sea ice cover change | Sea ice is projected to shrink in both the Arctic and Antarctic under all emission scenarios. In some projections, Arctic late-summer sea ice disappears almost entirely by the end of the 21st century. | | Consistent projection of decrease in Arctic and Antarctic sea ice cover in all models, although exact amount varies largely between emission scenarios and models | |
| Ocean circulation | Increases and decreases in ocean currents and ocean circulation patterns. Zero change to over 50% reduction in the Atlantic Ocean Meridional Overturning Circulation (MOC). | | It is considered very likely that the MOC will slow down during the course of the 21st century. Models consistently predict this although there is a large variation between models | |
| Wind fields and wind speed | Increases and decreases in mean wind speeds by 10-20% and changes in wind directions. Increased peak wind intensities and increased frequency of tropical storms. | | Large uncertainties in predictions and high spatial variability. | |

***Section S2: Simulating the global-scale fate and transport of contaminants for default and future climate scenarios using the BETR-IPCC model***

*Projected temperature and precipitation changes*: Projections for temperature and precipitation changes for the A1B scenario for the period 2080–2099 were summarized for 30 regions in the IPCC 4^th^ assessment report [2] on a seasonal and annual basis and then compared to the baseline period 1980–1999. The regions defined by the IPCC were used to revise the geographical segmentation of the BETR-World model subsequently applied here (BETR-IPCC, see Figure S1). The regional identifiers are presented in Table S2 and the projections for each region for the A1B scenario based on 21 models are presented in Table S3 (annual basis). Note that the ocean regions of the original BETR-World model were not revised when parameterising BETR-IPCC.

According to Christensen et al. [4], global mean near-surface temperature responses (i.e. baseline vs. 2080 – 2099) generated by these models are in the ratio of approximately 0.7:1:1.2 for the B1:A1B:A2 scenarios respectively. Near-surface temperature responses in the majority of IPCC regions also follow the same ratios. Mean local precipitation response generally scales with the global mean temperature response as well albeit with greater deviations. This is not surprising, given the greater variability/larger discrepancies in model output for precipitation response for a single scenario (see Table S3). All models display a linear response to the climate forcings *considered in the simulations* (i.e. non-linearities in response are not expected on regional/global scales but may occur in specific locations for e.g. in areas experiencing rapid loss of sea-ice). Note that in many regions, the expected differences between scenarios (0.7:1:1.2) are less than the observed differences between the minimum, median and maximum projection amongst models for a single scenario (see Table S3).

**Table S2.** IPCC regions and regional identifiers

| **Continent/Land mass** | **Region** | **Identifier** |
| --- | --- | --- |
| Africa  Europe & Mediterranean  Asia  North America  Central & South America  Australia & New Zealand  Polar Regions | West Africa  East Africa  South Africa  Sahara/North Africa  Northern Europe  Southern Europe & Med  North Asia/Siberia  Central Asia  Tibet  East Asia  South Asia  Southeast Asia  Alaska/Archipelago  Central sub-Arctic/Greenland  Western North America  Central North America  Eastern North America  Central America  Amazonia  Southern South America  North Australia  South Australia  Arctic  Antarctic | WAF  EAF  SAF  SAH  NEU  SEM  NAS  CAS  TIB  EAS  SAS  SEA  ALA  CGI  WNA  CNA  ENA  CAM  AMZ  SSA  NAU  SAU  ARC  ANT |

*Ocean regions are as follows: Northern North Atlantic (NNA), Southern North Atlantic (SNA), Northern South Atlantic (NSA), Southern South Atlantic (SSA), Northern Pacific West (NPW), Northern Pacific East (NPE), Northern South Pacific (NSP), Middle South Pacific (MSP), Southern South Pacific (SSP), Northern Indian Ocean (NIO), Southern Indian Ocean (SIO) and Southern South Indian (SSI)


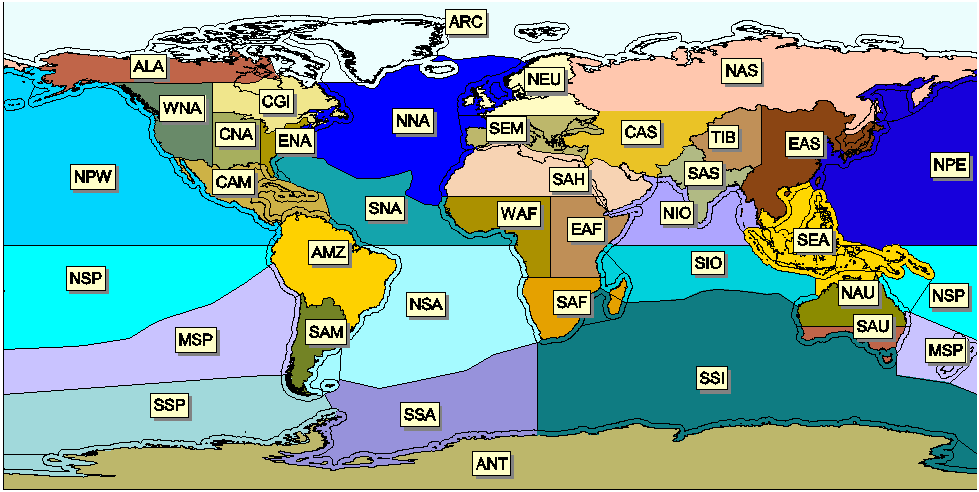


**Figure S1.** Map of BETR-IPCC with regional identifiers (See Table S2)

**Table S3.** Projected changes in annual average temperature (ΔT, ^o^C) and annual precipitation rate (ΔP, %) in IPCC regions summarized for the years 2080-2099 compared to the years 1980-1999 (baseline) of the A1B scenario. Projected values are based on output from 21 Atmosphere-Ocean General Circulation models (AOGCM)

| Identifier | Min 25 **50** 75 Max | Min 25 **50** 75 Max |
| --- | --- | --- |
| WAF  EAF  SAF  SAH  NEU  SEM  NAS  CAS  TIB  EAS  SAS  SEA  ALA  CGI  WNA  CNA  ENA  CAM  AMZ  SSA  NAU  SAU  ARC  ANT  CAR  IND  MED  TNE  NPA  SPA | 1.8 2.7 **3.3** 3.6 4.7  1.8 2.5 **3.2** 3.4 4.3  1.9 2.9 **3.4** 3.7 4.8  2.6 3.2 **3.6** 4.0 5.4  2.3 2.7 **3.2** 4.5 5.3  2.2 3.0 **3.5** 4.0 5.1  2.7 3.4 **4.3** 5.3 6.4  2.6 3.2 **3.7** 4.4 5.2  2.8 3.2 **3.8** 4.5 6.1  2.3 2.8 **3.3** 4.1 4.9  2.0 2.7 **3.3** 3.6 4.7  1.5 2.2 **2.5** 3.0 3.7  3.0 3.7 **4.5** 5.2 7.4  2.8 3.5 **4.3** 5.0 7.1  2.1 2.9 **3.4** 4.1 5.7  2.4 3.0 **3.5** 4.4 5.8  2.3 2.8 **3.6** 4.3 5.6  1.8 2.6 **3.2** 3.6 5.0  1.8 2.6 **3.3** 3.7 5.1  1.7 2.3 **2.5** 3.1 3.9  2.2 2.8 **3.0** 3.5 4.5  1.9 2.4 **2.6** 2.8 3.9  2.8 4.0 **4.9** 5.6 7.8  1.4 2.3 **2.6** 3.0 5.0  1.4 1.8 **2.0** 2.4 3.2  1.4 1.9 **2.1** 2.4 3.7  1.7 2.2 **2.7** 3.0 4.2  1.4 1.9 **2.0** 2.4 3.5  1.5 1.9 **2.3** 2.6 3.7  1.4 1.7 **1.8** 2.0 3.1 | -9.0 2.0 **2.0** 7.0 13.0  -3.0 2.0 **7.0** 11.0 25.0  -12.0 -9.0 -**4.0** 2.0 6.0  -44.0 -24.0 **-6.0** 3.0 57.0  0.0 6.0 **9.0** 11.0 16.0  -27.0 -16.0 **-12.0** -9.0 -4.0  10.0 12.0 **15.0** 19.0 25.0  -18.0 -6.0 **-3.0** 2.0 6.0  -1.0 2.0 **10.0** 13.0 28.0  2.0 4.0 **9.0** 14.0 20.0  -15.0 4.0 **11.0** 15.0 20.0  -2.0 3.0 **7.0** 8.0 15.0  6.0 13.0 **21.0** 24.0 32.0  8.0 12.0 **15.0** 20.0 31.0  -3.0 0.0 **5.0** 9.0 14.0  -16.0 -3.0 **3.0** 7.0 15.0  -3.0 5.0 **7.0** 10.0 15.0  -48.0 -16.0 **-9.0** -5.0 9.0  -21.0 -3.0 **0.0** 6.0 14.0  -12.0 -1.0 **3.0** 5.0 7.0  -25.0 -8.0 **-4.0** 8.0 23.0  -27.0 -13.0 **-4.0** 3.0 12.0  10.0 15.0 **18.0** 22.0 28.0  -2.0 9.0 **14.0** 17.0 35.0  -39.0 -19.0 **-12.0** -3.0 11.0  -2.0 3.0 **4.0** 5.0 20.0  -30.0 -16.0 **-15.0** -10.0 -6.0  -7.0 -3.0 **1.0** 1.0 7.0  0.0 3.0 **5.0** 10.0 19.0  -4.0 3.0 **3.0** 6.0 11.0 |

***Additional information on model parameterization.***

*Soil organic carbon.*A table listing the %OC in topsoil and subsoil were downloaded from the FAO GeoNetwork website (http://www.fao.org/geonetwork/srv/en/metadata.show?id=14116 ). In this table, data for major soil groups were listed (Ag vs Ag1) and linked to the FAO/UNESCO Digital Soil Map of the World (DSMW), a shapefile coverage of the major soiltypes of the world. The DSMW was intersected with the IPCC model regional map and the proportion of each IPCC region covered by each soil type was determined. Using a weighted average by area, the average topsoil organic carbon (%) in each IPCC region was determined. These values are presented in Figure S2 and summarized in Table S4.


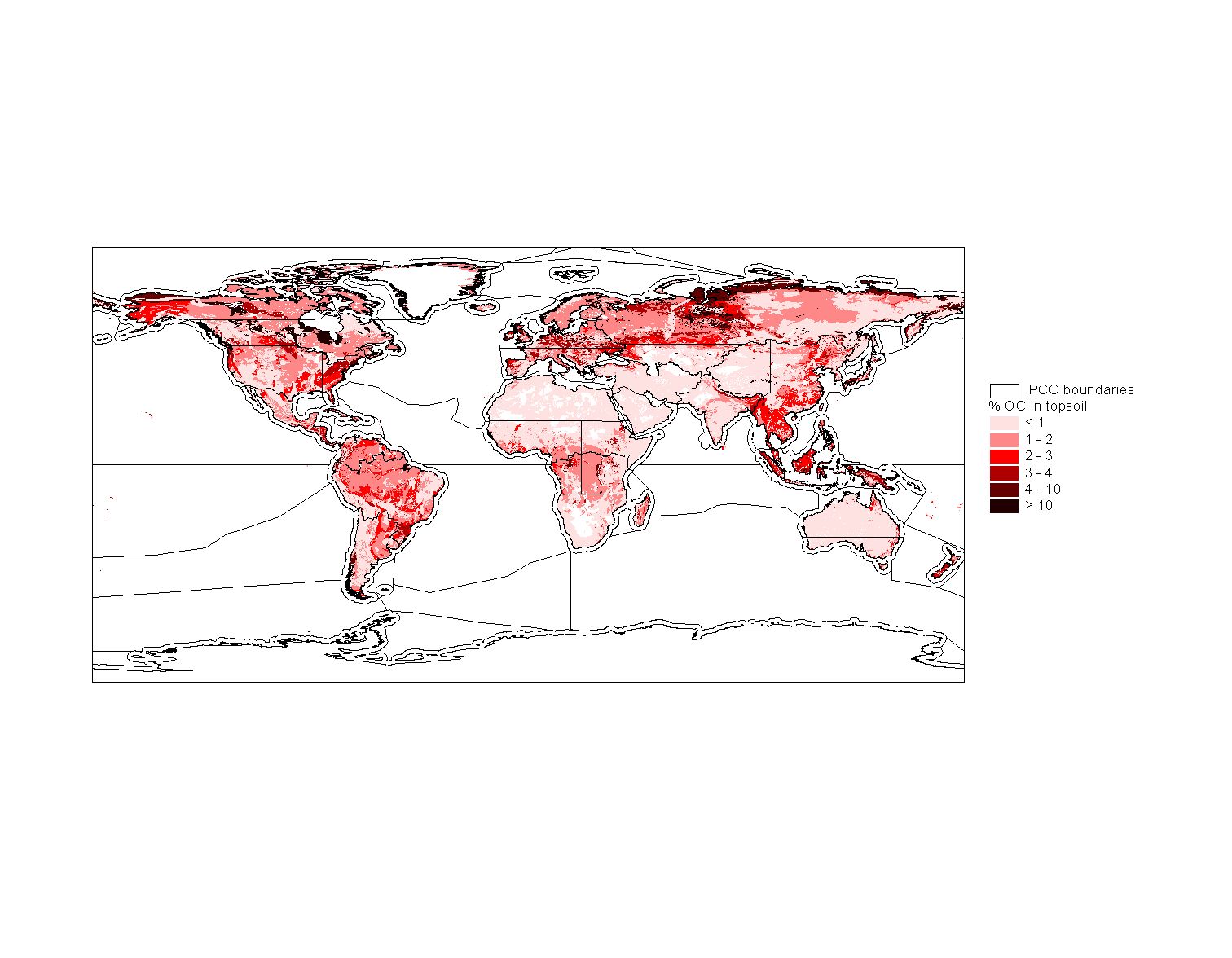


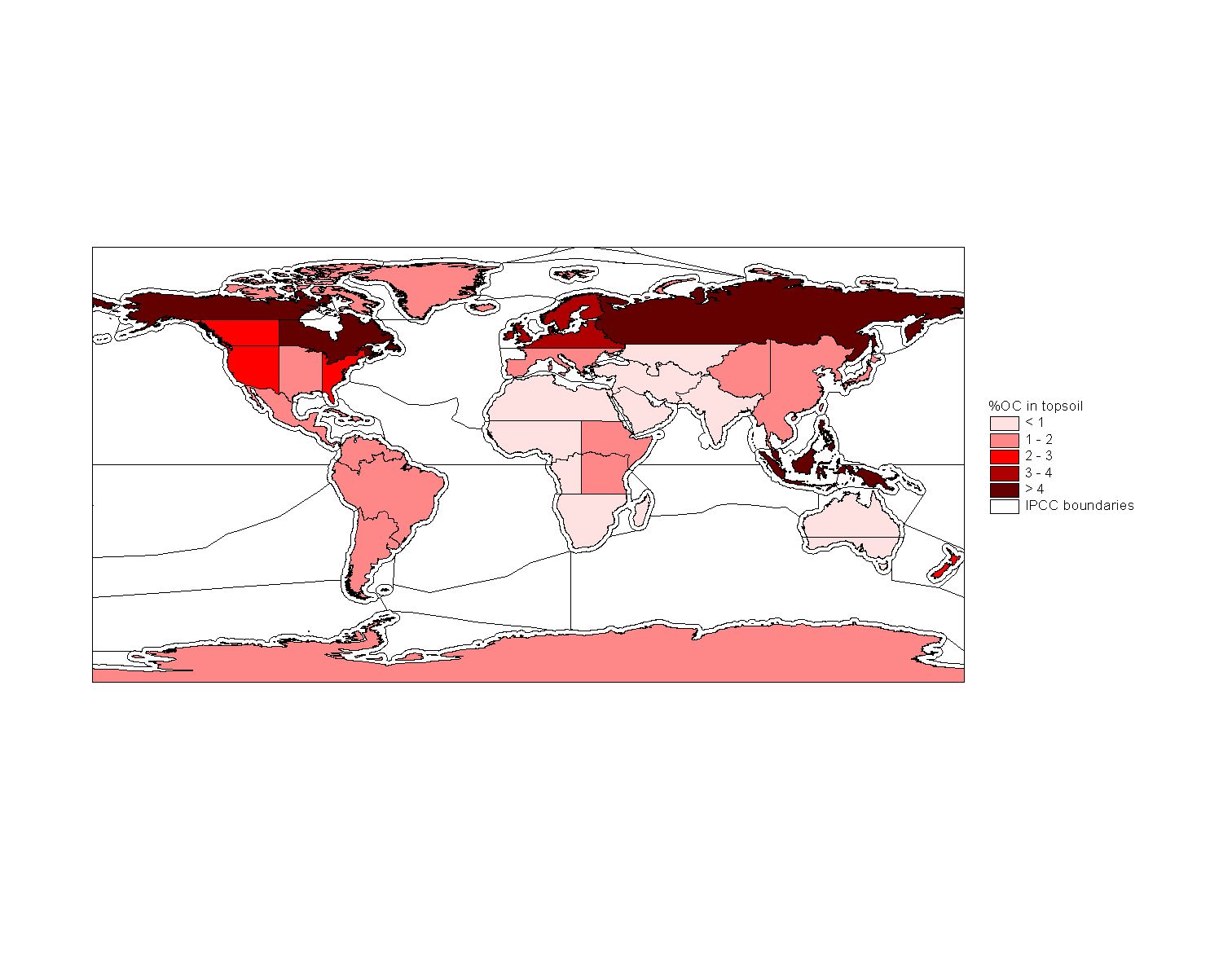


**Figure S2.** Global soil organic carbon content (topsoils) map (top panel) and weighted averages for the BETR-IPCC model regions (bottom panel)

**Table S4.** Revised soil organic carbon contents for continental regions in the BETR-IPCC model

| Identifier | Soil organic carbon content (fraction) | Identifier | Soil organic carbon content (fraction) |
| --- | --- | --- | --- |
| WAF  EAF  SAF  SAH  NEU  SEM  NAS  CAS  TIB  EAS  SAS  SEA | 0.010  0.011  0.007  0.004  0.036  0.015  0.041  0.009  0.015  0.015  0.009  0.056 | ALA  CGI  WNA  CNA  ENA  CAM  AMZ  SAM  NAU  SAU  ARC  ANT | 0.047  0.096  0.030  0.019  0.027  0.017  0.015  0.015  0.005  0.007  0.02  - |

No changes were made to average soil organic carbon contents for the future climate scenario as model projections of soil organic matter (SOM) indicate that only minor changes are to be expected on regional and global-scales [5,6]. This model behaviour reflects compensatory processing in the environment i.e. enhanced degradation of SOM due to warmer temperatures is counteracted by enhanced net primary productivity and subsequent deposition of OM to upper soil layers.

*Air Circulation Patterns.*

Region to region air circulation was characterized using the method described by Toose et al. (2004). Using the 2.5-degree Zonal and Meridonal Pressure Data from the NOAA NCEP/NCAR-CDAS (National Oceanic and Atmospheric Association - National Centers for Environmental Prediction/National Center for Atmospheric Research Climate Data Assimilation System) [7], a 50 year (1949-2001) annual average of wind direction at 850 mB for the lower air and 500 mB for the upper air compartments calculated for each 2.5-degree by 2.5-degree cell. Figure S3a,b shows calculated 50-year average global wind direction patterns for the approximate lower air (850mB) and upper air (500mB) heights.


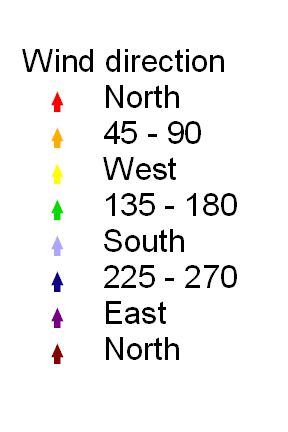
a)
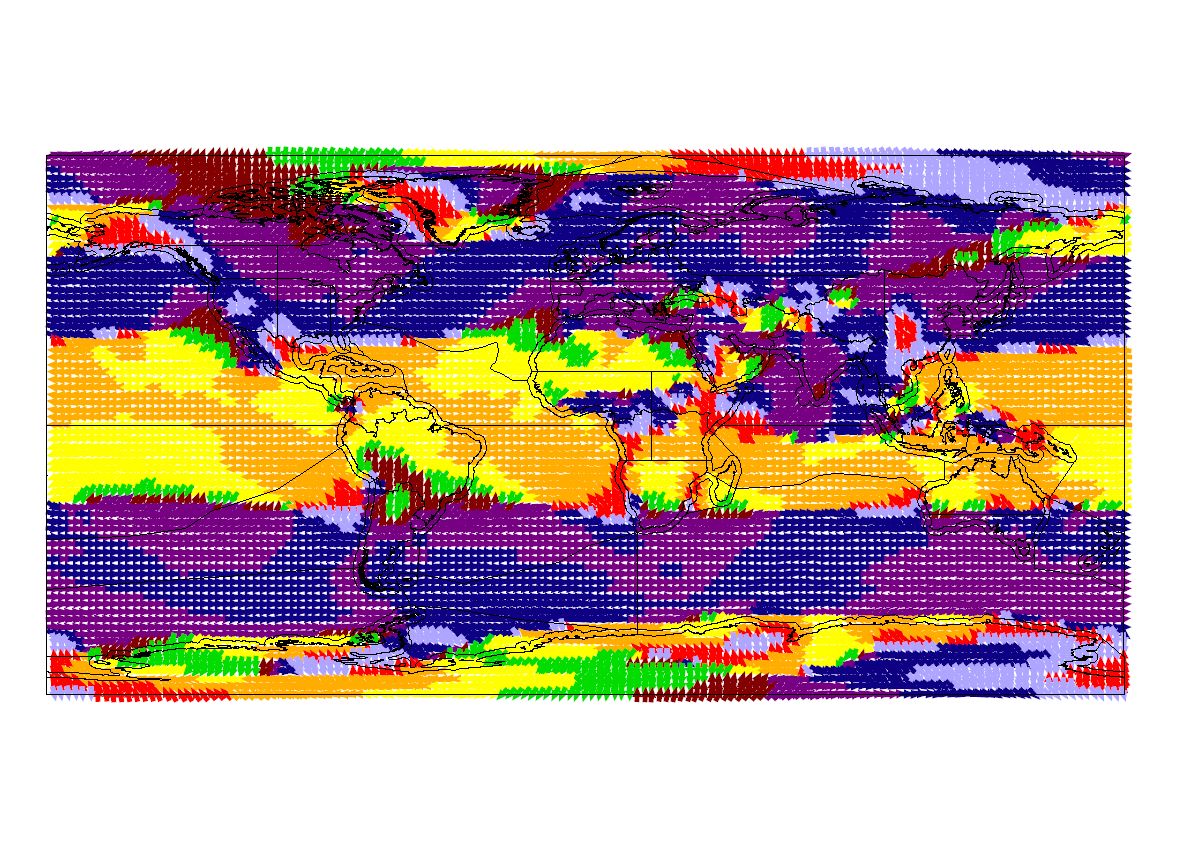


b)
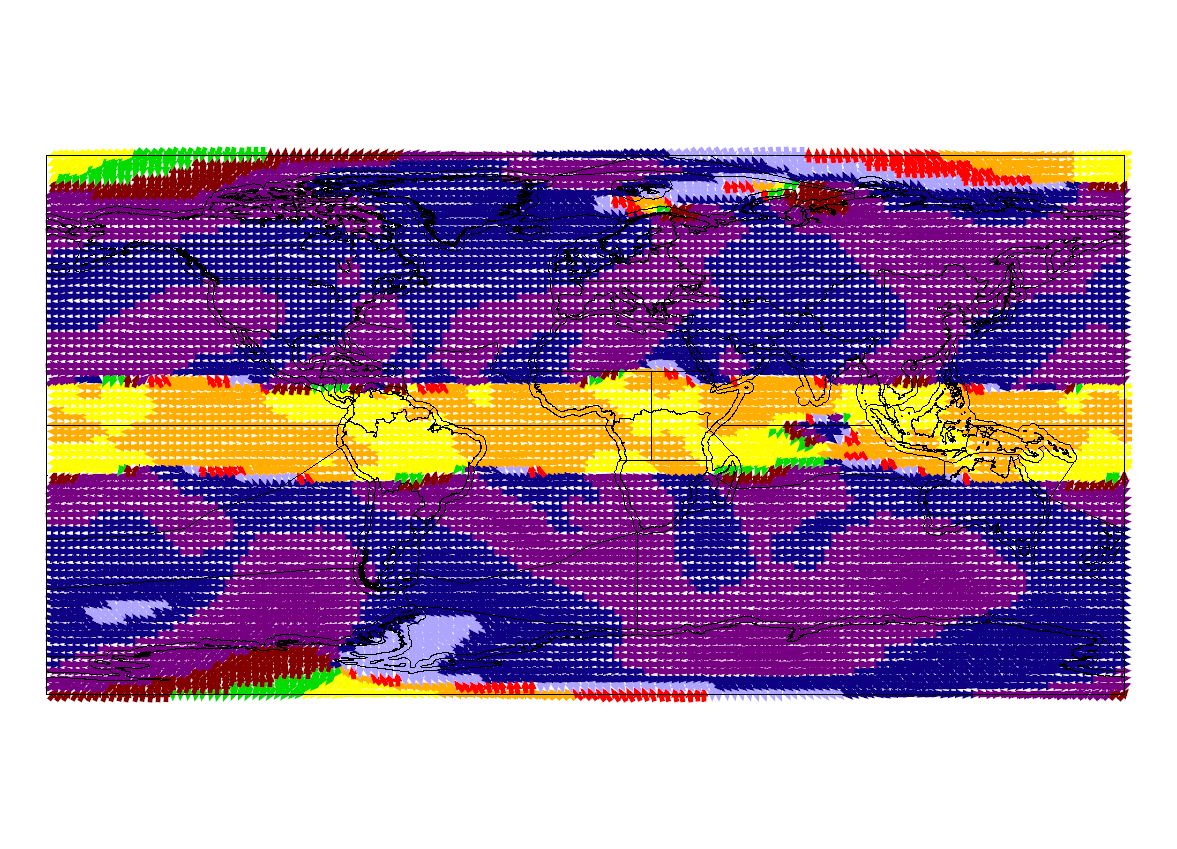


Figure S3a,b: Fifty year annual average wind direction for a) 850mB (lower air) and 500mB(upper air) (Graham et al. 1999).

At the boundaries of each region, the proportion of air leaving each region into adjacent regions was determined. For adjacent regions where there is strong directional flow into the target region, it was assumed that between 1-5% of the target region’s air volume flowed back into the source region. Airflow across the equator was restricted to 1-5% of each regional volume as well, giving hemispheric residence times of about 2-3.5 years for the lower air compartment and 0.5-1 year for the upper air compartment.

***Marine Circulation Patterns***

The marine regions are divided into coastal zones (within 200 km of landmasses) and oceanic zones and delineated according to major ocean currents as described in Toose et al. [8]. A five-degree interpolated marine surface drifter buoy dataset was obtained from the National Oceanic and Atmospheric Association, Physical Oceanography Division, Global Drifter Program Data Assembly Center [9]. Information regarding the average speed of buoys travelling north and east were used to calculate an average annual direction of surface flow for each datapoint. These data were then assessed in the same way as the atmospheric circulation data to obtain average flows between marine regions and into and out of the coastal portions of these regions.

*Runoff volumes.*

To estimate region-to-region continental runoff rates, a global 5-degree resolution coastal summary dataset characterizing the annual discharge of freshwater by rivers into the adjacent marine water body in each cell [10] was used. These cells were summarized by region and estimates of regional freshwater (and thus, chemical) input into adjacent marine regions were made.

**Model application – Hypothetical Chemicals**. Simulations were conducted for a set of hypothetical chemicals covering a wide range of partitioning property space. For the hypothetical chemicals, the partitioning property space was defined by conducting simulations with the octanol-air partition coefficient (log K_OA_ = 4 – 15) and air-water partition coefficient (log K_AW_ = -5 to 3). The octanol-water partition coefficient was calculated from the other two properties as shown below:

$$\log K_{OW}=\log K_{OA}+\log K_{AW}$$

Results are presented only for those combinations of log K_OA_and log K_AW_corresponding to log K_OW_ values between 1 and 12.

*Degradation.*Degradation half-lives (at 25^o^C) and activation energies (kJ/mol) of reaction for all hypothetical chemicals were as shown in Table S5.

**Table S5.** Degradation half-lives (at 25^o^C) and activiation energies (kJ/mol) assumed for all hypothetical chemicals

| Compartment | Half-life (h) | Activation Energy |
| --- | --- | --- |
| Air  Vegetation  Water (fresh, marine)  Soil  Sediments (fresh) | 480  1000  5500  17000  55000 | 10  30  30  30  30 |

*Temperature Dependence*.A key consideration of model parameterization, especially in the context of comparing model output under different climatologies, is the assumption for the temperature dependence of partitioning properties (i.e. internal energies of phase change, dU_ij_). The approach applied here is as follows:

Octanol-water partitioning (dU_OW_): All compounds were assumed to have dU_OW_ = -20 kJ/mol [11].

Octanol-air partitioning (dU_OA_): As shown in Table S6

Air-water partitioning: dU_AW_ = dU_OW_ – dU_OA_

**Table S6.** Assumed temperature dependence of the octanol-air partitioning coefficient

| log K_OA_  (at 25 ^o^C) | dU_OA_  (kJ/mol) | log K_OA_  (at 25 ^o^C) | dU_OA_  (kJ/mol) |
| --- | --- | --- | --- |
| 4  5  6  7  8  9 | -47  -55  -63  -71  -79  -87 | 10  11  12  13  14  15 | -95  -103  -111  -119  -127  -135 |

Note that the assumptions in Table S6 are based on the relationship between log K_OA_ (25^o^C) and estimated dU_OA_ for PCBs and other POPs for which data are available (for e.g. [12]). The accuracy of these assumptions across the full range of partitioning space considered is uncertain. However, given the available data, assuming the same temperature dependence across the entire partitioning space seems less appropriate.

*Primary Emissions.*Emissions were directed 100% to the lower atmosphere and distributed following the estimated spatial distribution of PCB 153 derived by Breivik et al. [13] for the year 1980 (see http://www.nilu.no/projects/globalpcb/). The fraction of total emissions assigned to each BETR-IPCC region is summarized in Table S7.

*Temperature and Precipitation Scenarios.*Results for all hypothetical chemicals were generated using the median temperature and precipitation rate changes shown in Table S3.

*Atmospheric and marine circulation patterns.*Lamon et al. [14] included alterations to annual average wind speed and oceanic circulation patterns in the GCC scenario (A2) developed for that modelling study. Changes in average circulation patterns were by far the least influential GCC change incorporated into the scenario. For example, in most regions of the model domain, model output generated under the GCC scenario incorporating changes only in atmospheric circulation was within ±10%. Given the low sensitivity of model output to alterations to both atmospheric and oceanic circulation patterns under steady-state assumptions, no GCC scenario for these parameters were developed for BETR-IPCC model application.

**Table S7.** Spatial distribution of emissions assumed for steady-state simulations

| Continent/Land mass | Identifier | Fraction of total emissions |
| --- | --- | --- |
| Africa  Europe & Mediterranean  Asia  North America  Central & South America  Australia & New Zealand  Polar Regions  Ocean Regions  (see above) | WAF  EAF  SAF  SAH  NEU  SEM  NAS  CAS  TIB  EAS  SAS  SEA  ALA  CGI  WNA  CNA  ENA  CAM  AMZ  SAM  NAU  SAU  ARC  ANT  (All) | 0.011  0.006  0.011  0.008  0.258  0.239  0.001  0.072  0.002  0.042  0.029  0.028  0.003  0.002  0.065  0.019  0.122  0.005  0.047  0.017  0.006  0.006  0.001  -  - |

**Model Application – PCB-153.**

Steady-state simulations were conducted for a PCB 153-like compound, following Lamon et al. [14], who also generated model output for a contemporary and future climate scenario (A2, 2080-2099) for PCB-like compounds.

*Partitioning properties and degradation half-lives.*

The partitioning properties and degradation half-lives selected for this application are shown in Table S8 and S9.

Table S8. Partitioning properties selected for the PCB-153 simulations

| Partition Coefficient | At 25 ^o^C | Internal Energy of Phase Change (kJ/mol) |
| --- | --- | --- |
| Octanol-air  Air-water  Octanol-water (calculated*) | 9.45  -2.13  7.31 | -94.8  68.2  -26.6 |

*log K_OW_ = log K_AW_ – log K_OA_; dry octanol basis; not adjusted to wet octanol

Table S9. Degradation half-lives selected for the PCB-153 simulations

| Compartment | Half-life (h) | Activation Energy |
| --- | --- | --- |
| Air  Vegetation  Water (fresh, marine)  Soil  Sediments (fresh) | *(region specific)**  55000  55000  55000  55000 | 10  30  30  30  30 |

*based on 2^nd^-order rate constant = 1.6e-13 cm^3^/molecule/s for reaction with OH radicals; adjusted to regional [OH]

*Temperature and Precipitation Scenarios.* Steady-state simulations of the PCB153-like compound were conducted assuming the median temperature and precipitation changes listed in Table S3 and also for a ‘worst-case’ scenario. For the ‘worst-case’ scenario, the maximum temperature changes listed in Table S3 were assumed while for precipitation either the minimum (if the median value indicated a precipitation decrease) or maximum (if the median value indicated a precipitation increase)values were used.

*Primary emissions and temperature.* Lamon et al. [14] suggested that the primary emissions of PCBs from certain sources (e.g. remaining stockpiles, landfills) occur via passive volatilization and hence are sensitive to ambient temperature changes. They proposed the following estimation approach to characterize this effect:

$$\frac{E_{2}}{E_{1}}=\exp\left[\frac{dU_{A}}{R}\cdot\left(\frac{1}{T_{1}}-\frac{1}{T_{2}}\right)\right]$$

where E_1_ and T_1_ are the baseline emission rate and ambient air temperature respectively, E_2_ and T_2_, are the emission rate and air temperature under the alternative scenario respectively, R is the gas law constant and dU_A_ is the internal energy of vaporization.

For the PCB153-like compound, a temperature increase of 4^o^C corresponds to emission rate ~ 1.6 - 1.7x higher (depending on the assumed baseline temperature).

**Model Output – PCB-153.**

*Comparison of model output for surface air concentrations: GCC vs Baseline.*Comparisons between steady-state surface air concentrations between the GCC scenarios and baseline are presented in Figures S4–S6. Figure S4 and S5 detail results for the median and maximum temperature and precipitation rate changes (no primary emission rate change) respectively while Figure S6 details the results for the median temperature and precipitation rate change including an increased primary emission rate.

Surface air concentrations in all regions are predicted to increase between 4 to 22% (average 13%) for the median climate scenario (Figure S4) and up to 32% (average 21%) for the maximum climate scenario (Figure S5). The largest increases in air concentration are predicted to occur in the Pacific marine regions and the Saharan, Antarctic and Eastern Canadian terrestrial (~ 20% in the median change climate scenario and ~ 30% under the maximum change climate scenario). While the warmer temperatures in the maximum GCC scenario correspond to higher steady-state air concentrations, the assumption regarding primary emission rate (↑ ~ 1.6x) has a far more important influence on model output (Figure S6).


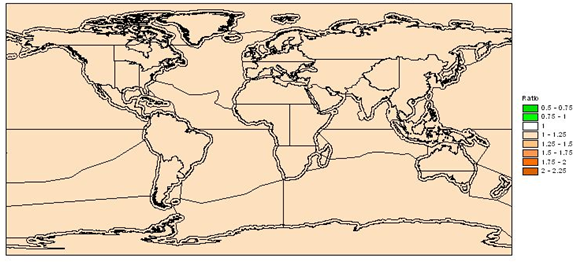


Figure S4. Comparison of steady-state surface air concentrations between the GCC scenario assuming median temperature and precipitation rate changes and the baseline with no change in primary emission rate


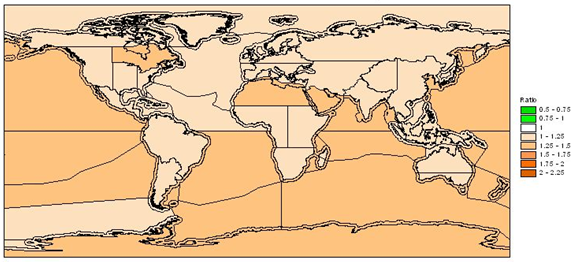


Figure S5. Comparison of steady-state surface air concentrations between the GCC scenario assuming maximum temperature and precipitation rate changes and the baseline with no change in primary emission rate


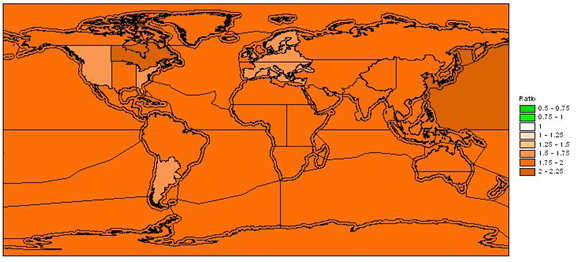


Figure S6. Comparison of steady-state surface air concentrations between the GCC scenario assuming mediantemperature and precipitation rate changes and the baseline, also assuming an increase in primary emissions

*Comparison of model output for total mass inventory: GCC vs Baseline.* Comparisons between steady-state surface total mass inventories between the GCC scenarios and baseline are presented in Figures S7–S9. Figure S7 and S8 detail results for the median and maximum temperature and precipitation rate changes (no primary emission rate change) respectively while Figure S9 details the results for the median temperature and precipitation rate change but also including increased primary emissions.

Total mass inventories in the median and maximum GCC scenarios (no change to primary emissions) decrease in all continental model regions (where emissions occur) compared to the baseline as does the total global mass inventory (all regions) (Figure S7, S8). The increased total mass inventories over oceans are driven by the increased air concentrations in these regions (see Figure S4, S5), reflecting enhanced transport from and reduced sequestration in source region reservoirs (e.g. surface soils). As expected, the increased primary emission rate counteracts the influence the temperature and precipitation scenario on total mass inventory (Figure S9); total mass inventories are increased in the GCC scenario in all zones except NAS and CGI (see Figure S1 for reference), where inventories are slightly higher in the baseline (1-2%).


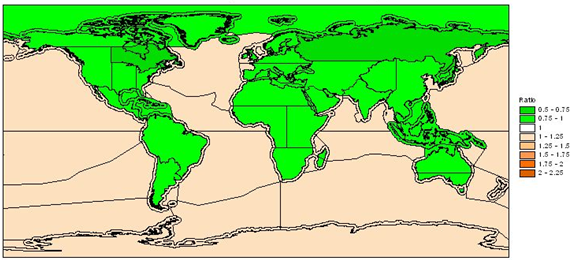


Figure S7. Comparison of steady-state total mass inventory between the GCC scenario assuming median temperature and precipitation rate changes and the baseline, assuming no change in primary emission rate


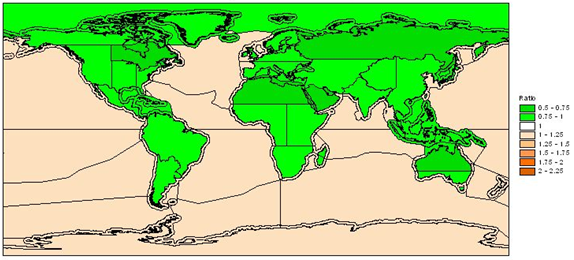


Figure S8. Comparison of steady-state total mass inventory between the GCC scenario assuming maximum temperature and precipitation rate changes and the baseline, assuming no change in primary emission rate


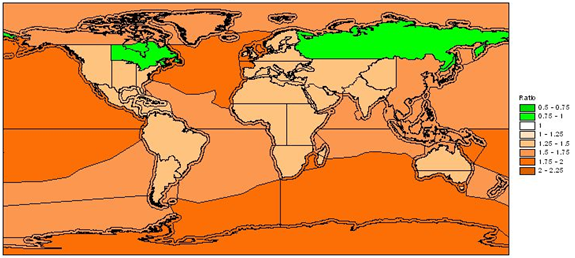


Figure S9. Comparison of steady-state total mass inventory between the GCC scenario assuming median temperature and precipitation rate changes and the baseline, also assuming an increasein primary emission rate

*Comparison of BETR-IPCC model output for a PCB153-like compound to Lamon et al. [14]. .*As the spatial resolution, emission pattern and climate change scenario (A2 vs. A1B) are not identical between the two modelling exercises, a quantitative comparison of the results is not appropriate. Nevertheless, the following commonalities are worth reiterating:

1. Assumptions regarding primary emission rate enhancements are more influential than the temperature and precipitation changes in influencing steady-state surface air concentrations
2. Warmer climate scenarios correspond to reduced overall persistence (P_OV_) in the global environment (by ~ 20-25%). These results imply a faster dissipation of contaminants/response once primary emissions have ended
3. Surface air concentrations are enhanced globally, somewhat more so in remote regions (no/low emissions) such as the Polar Regions and over oceans. These results partly reflect enhanced long-range transport but also redistribution to the air phase

The broad similarities between these model results are expected given the similarities in model approach (fugacity-based), segmentation (e.g. air, water, vegetation, soil, sediment compartments) and parameterization of key mass transfer coefficients (e.g. often based on generic values suggested in Mackay [15]). The main purpose in presenting these results is to increase transparency and also build confidence in the results generated for the hypothetical chemicals.

***Section S3. Bioenergetics-based bioaccumulation approach used to project possible GCC impacts on contaminant accumulation in aquatic organisms.***

Based on the previous work of [16], the bioaccumulation of a set of hypothetical chemicals with octanol-water partition coefficients (K_OW_) ranging from 0 to 8(on a log scale) and biotransformationhalf-lives ranging from 0.1 to 1000 days was simulated using a bioaccumulation model adapted from [17]. The impact of GCC was integrated by coupling the bioaccumulation model to a temperature-dependent bioenergetics model based on the metabolic rates of the round goby, *Apollonia melanostomus*, developed by [18]. The round goby is a highly adaptable forage fish that has invaded many freshwater environments in North America. It is a good candidate for such simulations for two primary reasons. First, its adaptability means that it tolerates a wide range of temperatures. Thus, it serves as a conservative example of the impact of GCC-mediated temperature increases on fish metabolic rates. Second, as a forage fish the round goby is tightly coupled to the environment yet still subject to bioaccumulation. Its prey includes primary consumers, which are most directly affected by changing concentrations and conditions in the environment, but its trophic level allows an additional degree of chemical amplification.

The models are integrated based on three fundamental descriptions: that of the temperature dependence of metabolic rates, namely consumption and respiration rates (Equations 1-4), the bioenergetics equation describing growth (Equation 5) and the chemical mass balance in a fish (Equation 6).

In ectothermic organisms like fish, metabolic rates are especially sensitive to temperature. As described by [19], this temperature dependence takes a three-phase form, with a period of increase, a thermal optimum, and a rapid decline beyond the critical thermal limit. Mathematically, this can be described by an equation with four key temperatures: the lower critical limit (T_1_ or CTL_Min_) the thermal optima representing the temperatures as which rates are maximized (T_2_ and T_3_) and the upper critical limit (T_4_ or CTL_Max_). K-values (K_1_ through K_4_, with values between 0 and 1) describe the proportion of the maximum rate achieved at each of these temperatures.

(Equation 1)

where

and

(Equation 2)

These temperature dependencies are built into the bioenergetics model for an organism through the respiration (R) and consumption (C) rates.

(Equation 3)

(Equation 4)

The bioenergetics model describes an organism’s growth through the balance of energy uptake and loss rates.

(Equation 5)

The growth rate of an organism can then be calculated by computing the balance of energy taken in from the diet (J_D_, dietary energy density, J/g) and used for maintenance such as respiration (based on J_O2_, the oxycalorific coefficient) and the energy density of the organism, describing the energetic costs of adding mass (J_F_, fish energy density, J/g). J_D_ and J_F_ can be calculated from the proportions of water, lipid, and non-lipid organic matter in an organism [16].

The uptake and loss rates can, in turn, be used within a chemical mass balance (bioaccumulation) model, thus linking the influence of temperature on a species metabolism to its rate of chemical uptake and depuration.

(Equation 6)

The temperature profiles used in our projections describe the annual temperature profile of Lake Erie, Laurentian Great Lakes, USA. The temperature cycle is set up so that day 1 of the simulation coincides with the first of May. These profiles are for the current Lake Erie conditions and projections assuming a 1, 2 or 3 degree Celsius increase in annual mean surface temperature (Equation 7).

Equation 7

where:

and .

A summary list of the variable definitions and parameter values used for the round goby bioenergetics-based-bioaccumulation model is provided in Table S10.

**Table S10.**Summary list of the variable definitions and parameter values used for the round goby bioenergetics-based-bioaccumulation model.

| Variable | Description | Value | Unit | Source |
| --- | --- | --- | --- | --- |
| 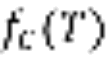 | Temperature dependence of consumption rate. | See Equation 1 | [ ] | (4) |
| T_1_ | Lower critical thermal limit for consumption. | 5.6 | °C | (3) |
| T_2_ | Lower optimal temperature for consumption. | 24.6 | °C | (3) |
| T_3_ | Upper optimal temperature for consumption. | 25.7 | °C | (3) |
| T_4_ | Upper critical thermal limit. | 29 | °C | (3) |
| K_1_ | Rate multiplier at T1. | 0.113 | [ ] | (3) |
| K_2_ | Rate multiplier at T2. | 0.98 | [ ] | (3) |
| K_3_ | Rate multiplier at T3. | 0.98 | [ ] | (3) |
| K_4_ | Rate multiplier at T4. | 0.419 | [ ] | (3) |
| 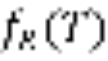 | Temperature dependence of respiration rate. | See Equation 2 | [ ] |  |
| C_Q_ |  | 0.061 | °C^-1^ | (3) |
| T_5_ | Upper critical thermal limit. | 0.01 | °C | (1) |
| K_5_ | Rate multiplier at T5. | 30 | [ ] | (1) |
| C | Consumption rate. | See Equation 3. | g/g/d |  |
| P | Fraction of maximum consumption realized. | 0.4 | [ ] | Based on (3)and observed growth rates for round gobies in Lake Erie. |
| a_1_ |  | 0.192 | [ ] | (3) |
| b_1_ |  | -0.256 | [ ] | (3) |
| W | Weight of fish. | 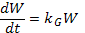 | g |  |
| R | Respiration rate. | See equation 4. | g/g/d |  |
| ACT | Activity coefficient. | 1.0 | [ ] | Assumes routine metabolism. |
| a_2_ |  | 0.00094 | [ ] | (3) |
| b_2_ |  | -0.157 | [ ] | (3) |
| k_G_ | Growth rate. | See equation 5. | g/g/d | (1) |
| J_D_ | Energy density of diet. | 2500 | J/g | (1) |
| J_O2_ | Oxycalorific coefficient. | 13600 | J/g | (3) |
| J_F_ | Energy density of round goby. | 4600 | J/g | (3) |
| SDA | Specific dynamic action. | 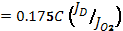 | g/g/d | (3) |
| F | Fecal egestion. | =0.15C | g/g/d | (3) |
| U | Excretion. | =0.085C | g/g/d | (3) |
| M_F_ | Mass of chemical in fish. | See equation 6 | g |  |
| k_W,in_ | Chemical uptake rate in fish from water. | =R×W/D_O2_ | L/d | Adapted from (2). |
| D_O2_ | Dissolved oxygen concentration of water. | =0.014×e^-0.02T^ | g/L | Assumes saturation. |
| C_w_ | Chemical concentration in water. | 1.0×10^-6^ | g/g | Assumed constant for simulations. |
| k_D_ | Dietary uptake rate. | =C×W | g/day | Adapted from (2). |
| C_D_ | Chemical concentration in diet. | =L_D_×K_OW_×C_W_ | g/g | Assumes equilibrium with water. |
| k_W,out_ | Chemical loss rate from fish to water. | =k_w,in_/K_OW_/L_F_ | g/d | Adapted from (2). |
| K_OW_ | Octanol-water partition coefficient for chemical. | log(K_OW_)=[0…8]  for simulations. | [ ] |  |
| L_F_ | Lipid content of fish. | 0.04 | [ ] | (3) |
| k_F_ | Rate of chemical loss from fish due to fecal egestion. | =0.15×k_D_×(L_D_/L_F_) | g/d | Adapted from (3)and (2). |
| L_D_ | Lipid content of diet. | 0.048 | [ ] | (1) |
| k_M_ | Rate of chemical biotransformation in fish. | =ln(2)/t_half_ | d^-1^ |  |
| t_half_ | Biotransformation half-life of chemical. | log(t_half_)=[-1to 3]  for simulations. | d |  |

We run the temperature-dependent bioenergetics-based bioaccumulation model at each combination of log(K_OW_) and t_half_. The simulations are run for five years, for four temperature regimes: current Lake Erie conditions, and a 1 through 3˚C increase. Finally, theconcentration ratios for the three temperature increase scenarios relative to the baseline (Lake Erie) scenario are computed over one season, during simulation year three(Figure 5C and 5D).

**Literature Cited**

**1. Nakicenovic N, Alcamo J, Davis G, de Vries B, Fenhann J, Gaffin S, Gregory K, Grubler A, Jung T, Kram T, La Rovere E, Michaelis L, Mori S, Morita T, Pepper W, Pitcher H, Price L, Riahi K, Roehrl A, Rogner H, Sankovski A, Schlesinger M, Shukla P, Smith S, Swart R, van Rooijen S, Victor N, Dadi Z. *Special report on emissions scenarios: A special reprot of working group III of the Intergovernmental Panel on Climate Change*. Cambridge University Press, Cambridge.**

**2. Pachauri R, Reisinger A. Climate Change 2007: Synthesis Report. Contribution of Working Groups I, II and III to the Fourth Assessment Report of the Intergovernmental Panel on Climate Change. IPCC, Geneva, Switzerland.**

**3. Moss RH, Edmonds JA, Hibbard KA, Manning MR, Rose SK, van Vuuren DP, Carter TR, Emori S, Kainuma M, Kram T, Meehl GA, Mitchell JFB, Nakicenovic N, Riahi K, Smith SJ, Stouffer RJ, Thomson AM, Weyant JP, Wilbanks TJ. 2010. The next generation of scenarios for climate change research and assessment. *Nature* 463:747-756.**

**4. Christensen J, Hewitson A, Busuioc A, Chen A, Gao X, Held I, Jones R, Kolli R, Kwon W-T, Laprise R, Magana Rueda V, Mearns L, Menendez C, Raisaneen A, Rinke A, Sarr A, Whetton P. Regional Climate Projections. In: Climate Change 2007: The Physical Science Basis. Contribution of Working Group 1 to the Fourth Assessment Report of the Intergovernmental Panel on Climate Change. Cambridge University Press, Cambridge.**

**5. Grace P, Post W, Hennessy K. 2006. The potential impact of climate change on Australia's soil organic carbon resources. *Carbon Balance and Management* 1:14.**

**6. King AW, Post WM, Wullschleger SD. 1997. The potential response of terrestrial carbon storage to changes in climate and atmospheric CO2. *Climatic Change* 35:199-227.**

**7. Kistler R, Kalnay E, Collins W, Saha S, White G, Woollen J, Chelliah M, Ebisuzaki W, Kanamitsu M, Kousky V, van den Dool H, Jenne R, Fiorino M. 2001. The NCEP-NCAR 50-year reanalysis: Monthly means CD-ROM and documentation. *Bulletin of the American Meteorological Society* 82:247-267.**

**8. Toose L, Woodfine DG, Macleod M, Mackay D, Gouin J. 2004. BETR-World: a geographically explicit model of chemical fate: application to transport of alpha-HCH to the Arctic. *Environmental Pollution* 128:223-240.**

**9. NOAA. DAC World data file: Mean velocity averages. NOAA Physical Oceanography Division, Global Drifter Program Data Assembly Center.**

**10. GRDC. BfG Freshwater fluxes into the World Oceans. BfG-Global Runoff Data Center.**

**11. Beyer A, Wania F, Gouin T, Mackay D, Matthies M. 2002. Selecting internally consistent physicochemical properties of organic compounds. *Environmental Toxicology and Chemistry* 21:941-953.**

**12. Schenker U, Macleod M, Scheringer M, Hungerbuhler K. 2005. Improving data quality for environmental fate models: A least-squares adjustment procedure for harmonizing physicochemical properties of organic compounds. *Environ Sci Technol* 39:8434-8441.**

**13. Breivik K, Sweetman A, Pacyna JM, Jones KC. 2007. Towards a global historical emission inventory for selected PCB congeners - A mass balance approach-3. An update. *Science of the Total Environment* 377:296-307.**

**14. Lamon L, von Waldow H, Macleod M, Scheringer M, Marcomini A, Hungerbuhler K. 2009. Modeling the Global Levels and Distribution of Polychlorinated Biphenyls in Air under a Climate Change Scenario. *Environ Sci Technol* 43:5818-5824.**

**15. Mackay D. 2001. *Multimedia environmental models: The fugacity approach*. CRC Press LLC, Boca Raton, FL.**

**16. Ng CA, Gray KA. 2011. Forecasting the effects of global change scenarios on bioaccumulation patterns in great lakes species. *Global Change Biology* 17:720-733.**

**17. Arnot JA, Gobas FAPC. 2004. A food web bioaccumulation model for organic chemicals in aquatic ecosystems. *Environmental Toxicology and Chemistry* 23:2343-2355.**

**18. Lee VA, Johnson TB. 2005. Development of a bioenergetics model for the round goby (Neogobius melanostomus). *Journal of Great Lakes Research* 31:125-134.**

**19. Thornton KW, Lessem AS. 1978. Temperature Algorithm for Modifying Biological Rates. *Transactions of the American Fisheries Society* 107:284-287.**
